# A novel luciferase-based assay for quantifying coronavirus-induced syncytia

**DOI:** 10.1038/s41598-025-02037-4

**Published:** 2025-05-20

**Authors:** Keisuke Oguma, Kenji Ogawa

**Affiliations:** 1 Laboratory of Veterinary Epizootiology, Department of Veterinary Medicine, College of Bioresource Sciences, Nihon University, 1866 Kameino, Fujisawa, 252-0880 Kanagawa Japan; 2 Drug Discovery Seeds Development Unit, RIKEN Center for Sustainable Resource Science (CSRS), 2-1 Hirosawa, Wako, 351-0198 Saitama Japan

**Keywords:** Syncytium quantification, Coronaviruses, Drug screening, SARS-CoV-2, Drug discovery, Tau proteins, High-throughput screening, SARS-CoV-2, Viral membrane fusion, Virology, Viral infection, Drug discovery

## Abstract

Coronaviruses can induce cell‒cell fusion that results in the formation of multinucleated syncytia through the interaction of viral spike proteins with host cell receptors. Quantifying syncytial formation is crucial for screening potential efficacious antiviral compounds. However, some traditional methods for syncytial quantification are often labor-intensive and limited by a low-throughput capacity. Therefore, we developed a novel high-throughput assay for the efficient quantification of syncytial formation induced by feline coronavirus (FCoV) and SARS-CoV-2. This assay, which is based on the split luciferase system, utilizes a split Gaussia luciferase (Gluc) system. In this system, fragments of Gluc are fused to the multimerizing Tau protein to reconstitute enzymatic activity upon cell fusion. In this study, the activity of the reconstituted luciferase was measured in 20 µL of culture medium to efficiently quantify syncytial formation induced by FCoV and SARS-CoV-2. Our findings demonstrate that this assay can accelerate the discovery of antiviral drugs that target coronaviruses.

**Supplementary Information:**

The online version contains supplementary material available at 10.1038/s41598-025-02037-4.

## Introduction

Several viruses, including paramyxoviruses^1,2^, retroviruses^3,4^, and coronaviruses^5–7^ enter host cells by fusing the viral envelope with the cell membrane. This fusion process is mediated by viral fusion proteins interacting with specific receptors on the host cell surface, consequently triggering a cascade of events that lead to viral infection^7–9^. The overexpression of viral fusion proteins can also induce cell‒cell fusion, mimicking the viral entry process^10–12^. The fundamental mechanism underlying cell fusion by viral proteins is similar to that of fusion between the viral envelope and the cell membrane; this mechanism involves interactions between fusion proteins and host cell receptors. Therefore, an efficient method for quantifying syncytial formation is required to develop compounds that inhibit viral entry and prevent infection.

Although established methods such as manual counting and fluorescence intensity measurements using microscopy or flow cytometry have provided valuable insights into viral fusion^13–16^, these approaches can be time-consuming and may not be ideal for high-throughput applications. In addition, detached syncytia present challenges for large-scale screening of antiviral compounds and detailed studies of viral fusion dynamics. Therefore, we developed a novel high-throughput assay to quantify syncytial formation induced by coronaviruses, specifically feline coronavirus (FCoV) and SARS-CoV-2. The developed assay is a rapid, convenient, and high-throughput tool for investigating viral pathogenesis, with the potential to accelerate the development of antiviral drugs.

## Results

### Restoration and detection of split-Gluc activity in culture media

**Fig. 1:**
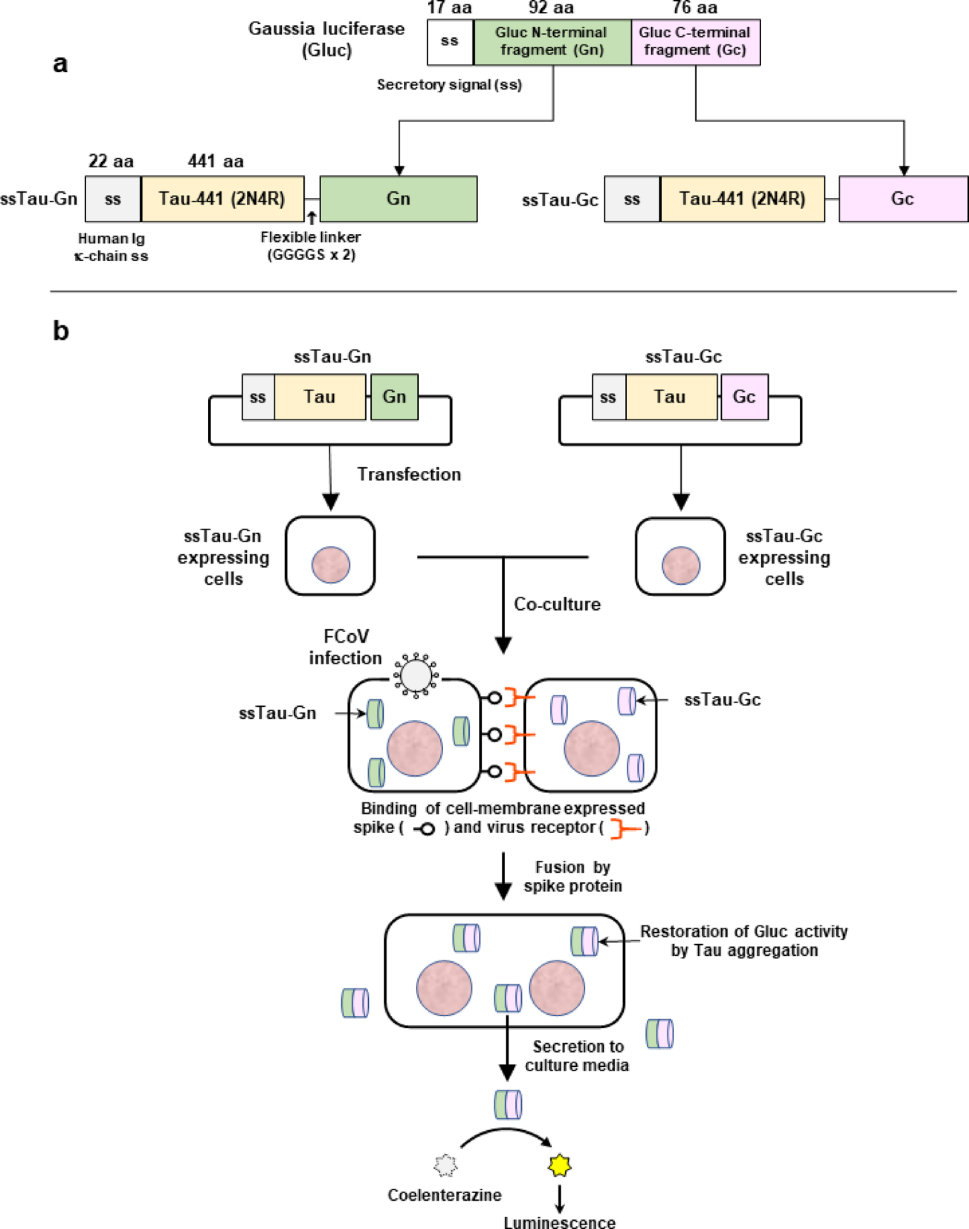
Overview of the ssTau-split Gluc system. (**a**) Structure of the ssTau-split Gluc protein. (**b**) ssTau-split Gluc expression vectors were independently introduced into the cells, which were then co-cultured at a 1:1 ratio. During coronavirus infection, spike proteins expressed on the cell membrane bind to the virus receptor to facilitate cell‒cell fusion. The aggregation of Tau proteins functionally restores Gluc activities that is released into the culture medium, enabling high-throughput measurement of Gluc activity without cell lysis.

In the present study, we employed an extracellularly secreted form of Tau-split *Gaussia* luciferase (Gluc) to quantify syncytial formation. This system relies on the restoration of split Gluc activity upon fusion of co-cultured cells expressing the secretory signal (ss)-tagged Tau-Gn (N-terminal fragment of Gluc) and ss-tagged Tau-Gc (C-terminal fragment of Gluc) (Fig. 1a). Multimerization of Tau fused with Gn or Gc allows the Gluc fragments to interact and restore luciferase enzymatic activity^17^ (Fig. 1b). Although Tau-mediated split Gluc restoration has been described in the study of the biochemical characteristics of Tau protein in neurodegenerative diseases^17^, its application to the detection and quantification of viral cell fusion is novel. We hypothesized that if split-Gluc enzymatic activity were restored only upon cell fusion, the resulting luciferase activity would serve as a direct indicator of these events. As a preliminary experiment, Gn and Gc fragments were fused to the N-terminus (ssGn-Tau and ssGc-Tau) and C-terminus (ssTau-Gn and ssTau-Gc) of the Tau protein. This generated four distinct fusion proteins for intracellular expression.

**Fig. 2:**
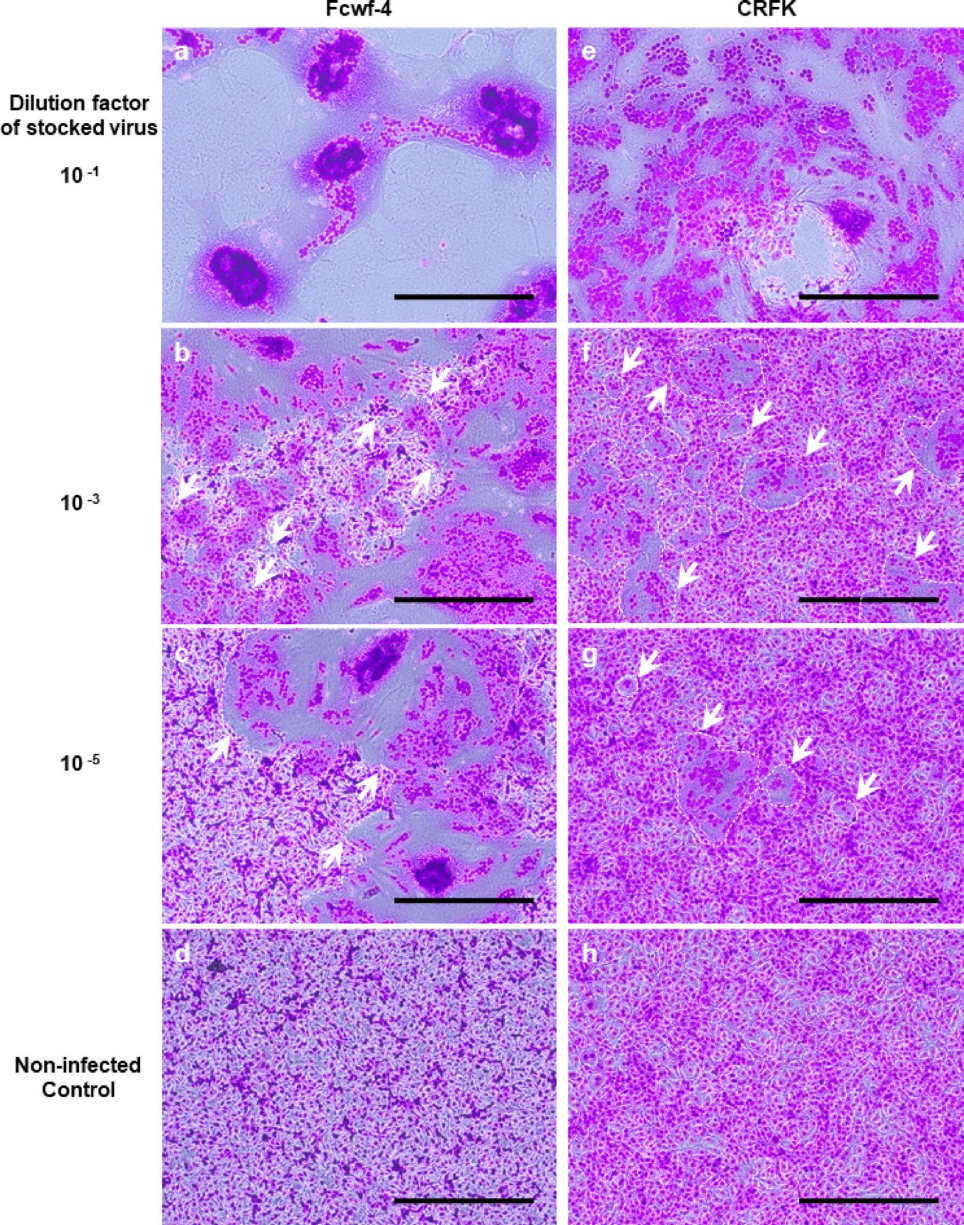
FCoV-induced cell fusion. Fcwf-4 (left panels, **a**–**d**) and CRFK (right panels, **e–h**) cells were cultured in 1 mL of medium per well in a 12-well plate. FCoV strain M91-267 stock virus was half-log diluted from 10^− 1^ to 10^− 6^ using culture medium and 0.5 mL of diluted virus were used for infection in a total 1 mL of medium/well. The cells were cultured for 18 h, fixed with methanol and stained with Giemsa solution. Both cell lines infected with virus diluted to 10^− 1^ had fused or detached cells throughout the well (panel **a** and **e**, respectively). Syncytia were surrounded by dashed line and some of them were indicated by arrows (panel **b**, **c**, **f**, and **g**). In panel (**b**), dashed line-surrounded areas indicate small regions consisted of non-syncytial cells. Scale bar: 200 μm.

When two feline culture cell lines—*Felis catus* whole fetus (Fcwf-4)^18^ and Crandell-Rees feline kidney (CRFK)^19^ cells—were infected with FCoV, they formed syncytial cells at varying degrees in number and size depending on virus dose (Fig. 2). Syncytia appeared larger and more abundant with higher virus doses. However, they were difficult to count when cells throughout the well were fused or detached, or when two more syncytia fused at the time of observation. Consequently, it is difficult to quantify syncytial cells through methods based on optical observation of stained cells, or by counting the numbers of syncytia emitting fluorescence in response to cell-fusion with a microscope or a flow cytometer. Therefore, the efficiency of our novel assay employing ssTau-split Gluc in syncytial quantification was validated using Fcwf-4 and CRFK cells.

**Fig. 3:**
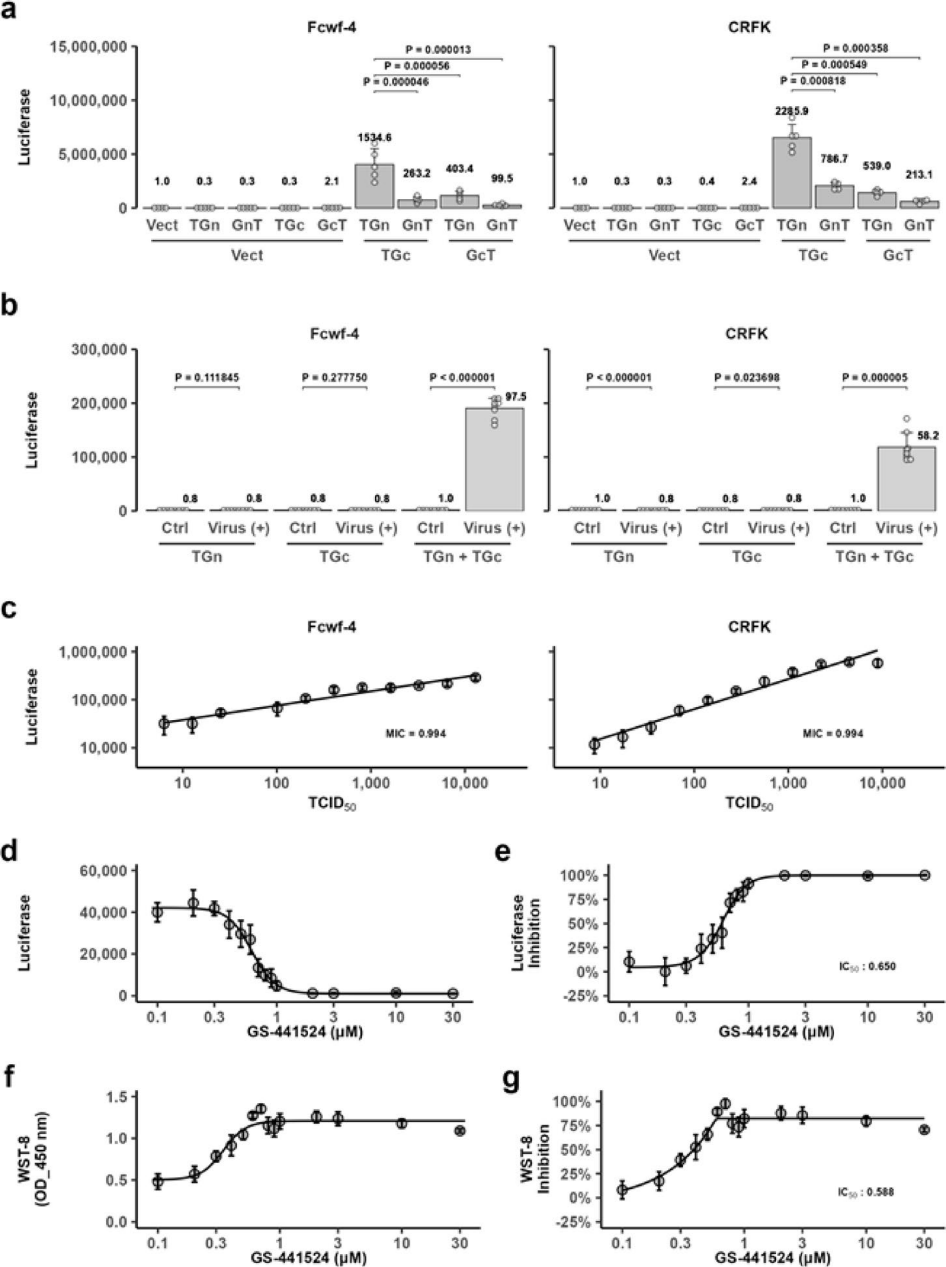
Upregulation of the ssTau-split Gluc signal via FCoV infection. (**a**) Gluc activity in the medium of cells transfected with a Gn/Gc pair of four Tau-split Gluc constructs (a split Gluc fragment (Gn or Gc) at the N- or C-terminus of Tau) in Fcwf-4 and CRFK cells. (1) Vect: Empty expression vector, (2) TGn: ssTau-Gn, (3) TGc: ssTau-Gc, (4) GnT: ssGn-Tau, (5) GcT: ssGc-Tau. The data are expressed as the mean ± standard deviation (SD), with raw values of each well indicated as circles (○). The luciferase values relative to cells transfected only with empty vectors are indicated at the top of each bar. (**b**) Quantification of FCoV-induced cell‒cell fusion using the ssTau-split Gluc system at 24 h post-infection. The transfected vectors and viral infections in each group are indicated in bar graphs. Ctrl: Non-infected control cells. The data are expressed as the mean ± SD, with raw values of each well indicated as circles. The luciferase values relative to non-infected cells with TGn + TGc vector are shown at the top of each bar. (**c**) Correlation between viral dose and split-Gluc restoration. The mean luciferase value of each group was used for the analysis. The data are expressed as the mean ± SD, with raw values of each well indicated as circles. MIC: Maximal information coefficient^20^. (**d**) Effects of GS-441524 on FCoV-induced syncytial formation of Fcwf-4 cells. Luciferase activity at various GS-441524 doses is shown. The data are expressed as the mean ± SD. (**e**) Inhibition of split-Gluc restoration by GS-441524. The percentage of inhibition was calculated based on the luciferase activity results shown in panel **d**. The data are expressed as the mean ± SD, with raw values of each well indicated as circles. (**f**) Effects of GS-441524 on cell survival. Cell viability was assessed using the WST-8 assay after the determination of split-Gluc values. The data are expressed as the mean ± SD, with raw values of each well indicated as circles. (**g**) Inhibition of cell death by GS-441524. The percentage of inhibition was calculated based on the WST-8 assay results shown in panel (**f**).

Although individual expression of the four vectors did not restore split Gluc activity, co-expression of ssTau-Gn and ssTau-Gc yielded the highest Gluc activity among the tested pairs (Fig. 3a). This split-luciferase system was further studied to evaluate its efficiency in the quantification of cell fusion. Infection of Fcwf-4 and CRFK cells with FCoV significantly increased split Gluc activity (Fig. 3b). Importantly, the co-culture of cells expressing either ssTau-Gn or ssTau-Gc alone did not increase luciferase activity in the absence of infection. Furthermore, the split Gluc activity strongly correlated with the infectious dose of FCoV (Fig. 3c). The maximal information coefficient, a novel and robust correlation index^20^ suitable for non-linear relationships, was 0.994 between the viral dose and split Gluc activity in both cell lines. Cells transfected with the ssTau-split Gluc vector and cultured for 48 h before use in FCoV infection experiments exhibited cell rounding, potentially due to the toxicity of overexpressed Tau protein.

Cells expressing ssTau-Gn and ssTau-Gc were co-cultured at varying ratios, ranging from 10:0 to 0:10, to determine the optimal co-culture ratio. A ratio of 1:1 showed the highest or second highest absolute luciferase values, with the highest relative luciferase values observed in virus-infected cells compared to non-infected control. In contrast, the ratio between 7:3 and 3:7 resulted in a > 60-fold and 30-fold increase in Fcwf-4 and CRFK cells, respectively (Supplementary Fig. 1a). Centrifugation (10,000 × *g*, 1 min) of the culture supernatants did not significantly reduce luciferase activity (Supplementary Fig. 1b). These results confirmed that luciferase was released into the culture media upon cell fusion and was not associated with luciferase-containing cell debris resulting from the cytopathic effect of FCoV and suspended in the supernatant.

Two-fold serial dilution of cells before FCoV infection indicated that using 5 × 10^4^ cells/100 µL/well in a 96-well plate is sufficient for luciferase detection. Additionally, 2.5 × 10^4^ cells/100 µL/well resulted in a > 10-fold increase in luciferase activity compared to the control (Supplementary Fig. 1c). Thus, a two-fold serial dilution was performed using culture media from FCoV-infected cells to assess the dynamic range of luciferase activity. The average luciferase activity decreased with linearity, going from approximately 95.1-fold and 55.4-fold of non-infected control in Fcwf-4 and CRFK cells, respectively, to 1.8-fold and 1.2-fold by 128-fold dilution of non-diluted culture media of non-infected cells (Supplementary Fig. 1d).

The application of our assay to a 384-well plate was also analyzed by determining luciferase activity in 20 µL of SUP samples in 100 µL of medium/well in a 96-well plate, as well as that of 50 µL/well or 100 µL/well in 384-well plate. Fcwf-4 and CRFK cells were cultured at 0.16–5 × 10^4^/well. Although this was a preliminary study, luciferase levels in the supernatants significantly increased not only in high-density wells, but also even in low-density wells seeded with 0.16 × 10^4^ cells/well (FCoV-infected Fcwf-4 cells) and 0.31 × 10^4^ cells/well (FCoV-infected CRFK cells) in 384-well plates (Supplementary Fig. 2a).

However, transferring supernatants to a separate measurement plate is laborious, costly, and impractical in a high-throughput screening setting using 384-well plates. Therefore, we verified that luciferase could be measured by dispensing coelenterazine directly into 384-well cell culture plates. To establish this direct measurement, Fcwf-4 cells were cultured for 24 h with or without FCoV in 10–50 µL of medium, with 5 µL of 10^− 1^-diluted FCoV added per well to maintain a consistent viral dose across all cell-densities. Fifty microliters of coelenterazine buffer was then directly dispensed into the 384-well white-colored tissue culture plate. Luciferase activity increased 3.6-fold compared to non-infected cells, even when cells were cultured at a low density of 0.16 × 10^4^ cells/well in 10 µL of culture medium (Supplementary Fig. 2b, top panel). The luciferase levels of FCoV-infected cells increased in proportion to cell-density and were inversely proportional to culture medium volume. However, those of non-infected cells cultured in 10 µL of medium also increased in proportion to cell density, resulting in the lowest signal/background ratio at 5 × 10^4^ cells/well. Similar results were obtained when the virus concentration, rather than the viral dose, was kept constant across different cell densities to eliminate the possibility that virus dilution with increasing medium volumes in each well could lead to lower luciferase values (Supplementary Fig. 2b, bottom panel).

To further validate the applicability of ssTau-split Gluc system, we assessed the inhibitory effects of the nucleoside analog GS-441524 on FCoV-induced syncytial formation and simultaneously monitored cell survival. The increase in luciferase activity was significantly suppressed in a dose-dependent manner (Fig. 3d and e). Furthermore, cell survival was inversely correlated with split-luciferase activity (Fig. 3f and g). Nucleoside analogs primarily target viral genome replication rather than directly inhibiting viral attachment or entry. Therefore, their inhibitory effect may be attributable to the suppression of viral protein expression, including that of spike proteins. Accordingly, we used a spike protein-induced cell fusion experiment to further validate this hypothesis.

### Spike-mediated cell-fusion assay

**Fig. 4:**
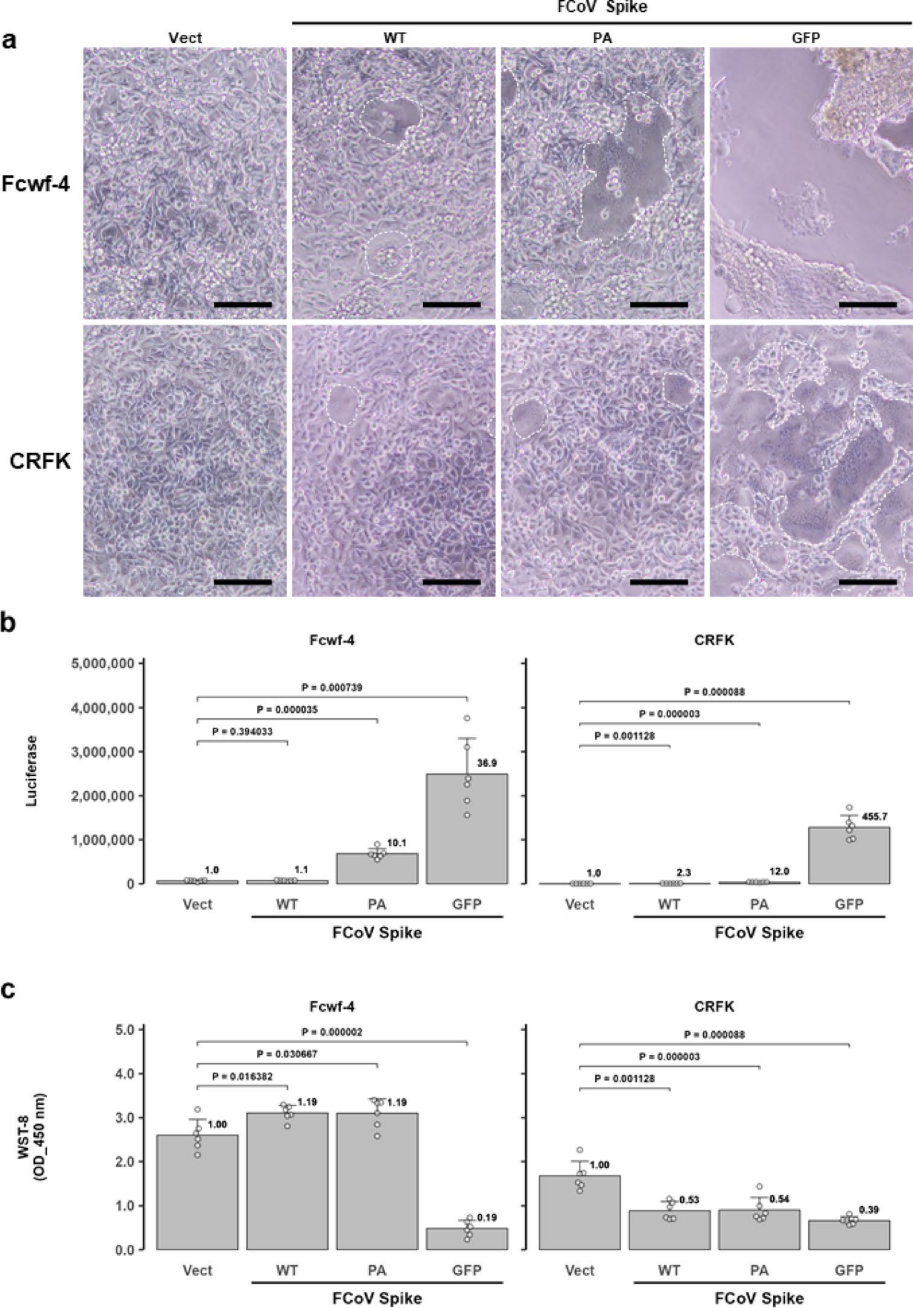
Quantitative analysis of FCoV spike protein-induced syncytia. (**a**) Light microscopy images of parental Fcwf-4 and CRFK cells transfected with FCoV S vectors and either ssTau-Gn or ssTau-Gc, as obtained 24 hpt. Syncytial regions are surrounded by dashed circles or lines. The arrows indicate syncytial cells. Nearly all Fcwf-4 cells expressing GFP-tagged spike formed syncytia in the culture well. Scale bar: 200 μm. (**b**) Quantification of FCoV spike protein-induced syncytia using the ssTau-split Gluc system. The data are expressed as the mean ± SD, with raw values of each well indicated as circles. The luciferase values relative to those of the empty vector control (Vect) are indicated at the top of each bar. (**c**) Cell survival analysis. Cell viability was assessed using the WST-8 assay after the determination of Gluc activity (panel **b**).

Fcwf-4 and CRFK cells were transiently transduced with an expression vector containing the FCoV S gene, along with either ssTau-Gn or ssTau-Gc. The S expression plasmid was transfected into the cells together with ssTau-split Gn or ssTau-Gc vector at a 10:1 ratio in micrograms. The restoration of Gluc activity was then measured at 24 h post-transfection (hpt). Although expression of the wild-type S protein induced minimal syncytial formation, the GFP (238 amino acids)-tagged spike protein (S-GFP) exhibited a significantly stronger capacity to induce cell‒cell fusion. This resulted in the formation of large syncytia that spontaneously detached from the culture wells (Fig. 4a and b).

Detachment occurred earlier in Fcwf-4 cells than in CRFK cells. Moreover, the cell fusion activity increased with the length of the tag protein or peptide. The S protein tagged with a shorter PA tag (12 amino acids)^21^ moderately increased the split Gluc activity. Thus, the increase in ssTau-split Gluc activity was directly proportional to the syncytia-inducing ability of the spike proteins, and cell survival was inversely correlated with luciferase activity (Fig. 4c).

Time-course analysis was performed using Fcwf-4 and CRFK cells. These cells were transfected with S-GFP for 2 h, trypsinized and collected, frozen once, thawed, and cultured in 96-well plates to analyze luciferase activity in the culture supernatant at 5, 6, 8, 10, 12, and 28 hpt. Although luciferase activity in the supernatant of Fcwf-4 cells expressing S-GFP began to increase at 12 hpt, the increase was only 2.7-fold compared to that in non-infected cells. Luciferase was first detected in CRFK cells after 28 hpt (Supplementary Fig. 3).

**Fig. 5:**
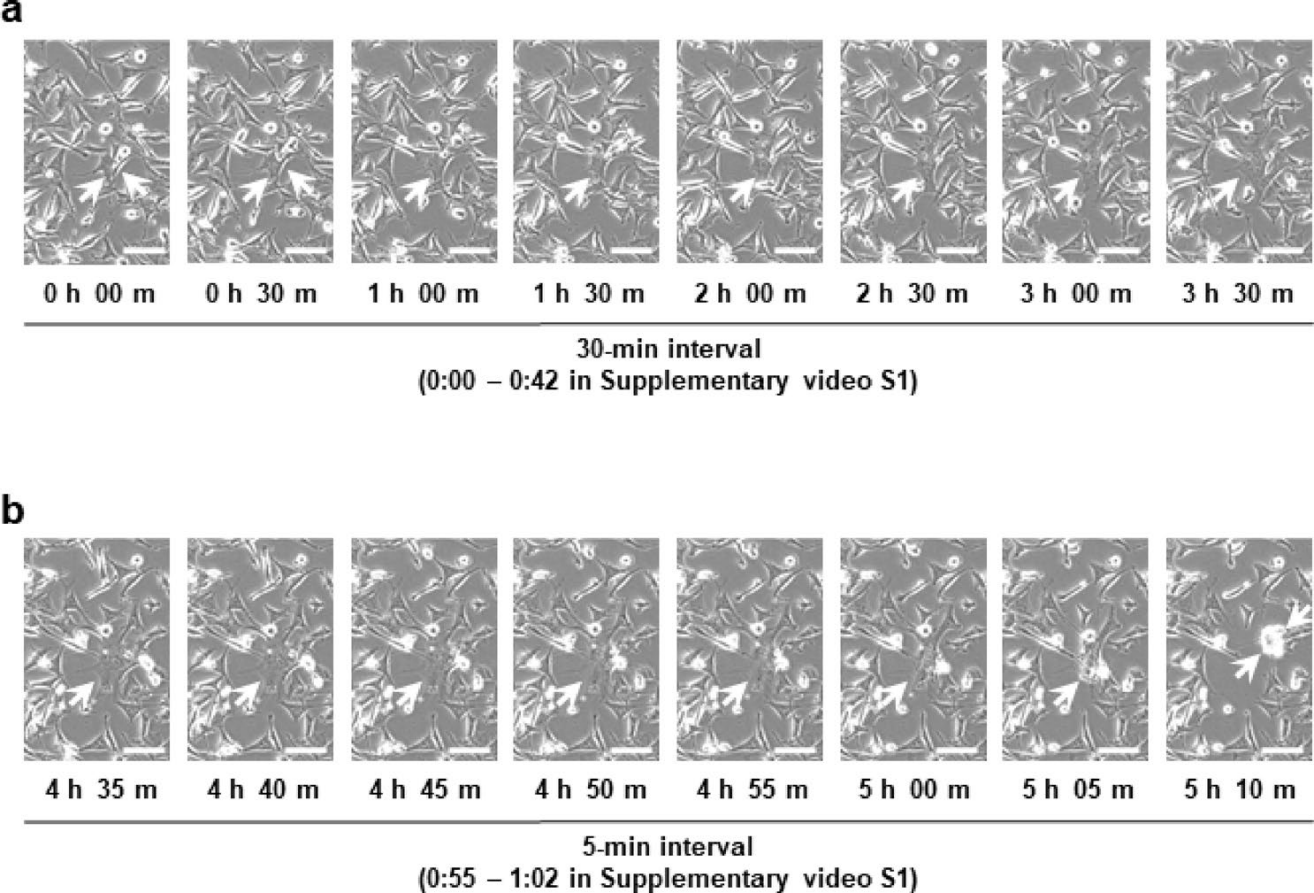
Time‒lapse microscopy of syncytial cell formation. (**a**) Early stages of syncytium formation in two parental Fcwf-4 cells transfected with the FCoV S-GFP vector. The initiation of fusion and syncytium formation are indicated by arrows. Images were presented at 30-min intervals. Scale bar: 100 μm. (**b**) Detachment of the syncytium. The syncytium shown in panel A detached from the culture well within 10 min (indicated by arrows in Panels 5 h 00 m to 5 h 10 m). Images were presented at 5-min intervals. Scale bar: 100 μm.

Time-lapse microscopy revealed a dynamic syncytium formation process. Two Fcwf-4 cells expressing the FCoV S-GFP fused and progressed into a small multinucleated cell within approximately 1 h. The resulting syncytia continued to grow, becoming larger over an additional 2.5 h, before detaching from the substrate (Fig. 5 and Supplementary Video S1).

**Fig. 6:**
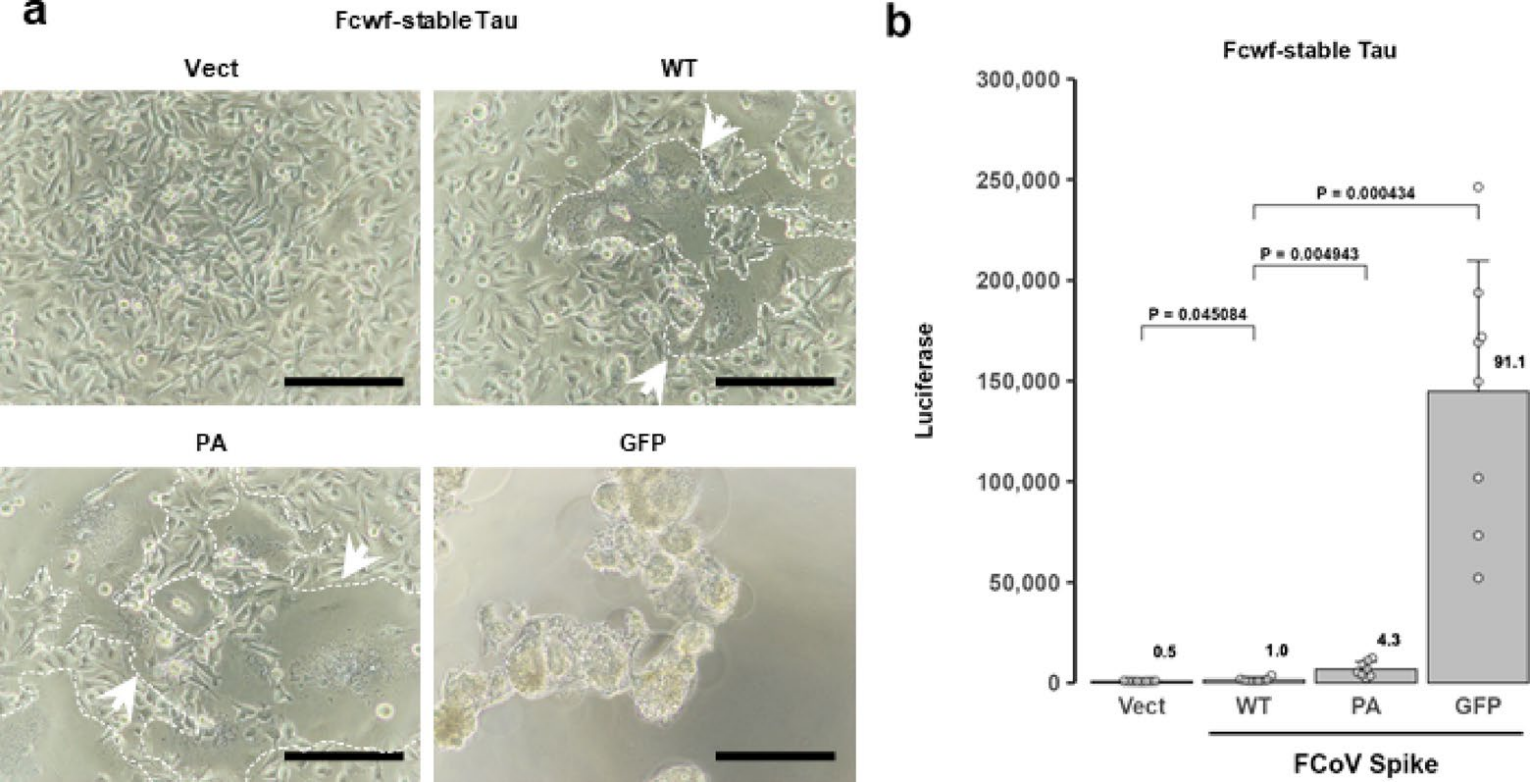
Quantitative evaluation of FCoV spike protein-induced syncytia in Fcwf-4 cells stably expressing ssTau-split Gluc. (**a**) FCoV S-induced syncytia in Fcwf-stable Tau cells. Fcwf-stable Tau cells were transfected with empty (Vect), tag-free (WT), PA-tagged, or GFP-tagged S expression vectors. Syncytia are surrounded by a dashed line in light microscopy images obtained 24 hpt. Scale bar: 100 μm. (**b**) Gluc activity of the cells shown in panel a, as determined 24 hpt. The data are expressed as the mean ± SD, with raw values of each well indicated as circles. The luciferase values relative to those of WT are indicated at the top of each bar.

Syncytium-inducing activity was further evaluated in Fcwf-4 cells stably expressing ssTau-Gn or ssTau-Gc (hereafter referred to as “Fcwf-stable Tau cells”), which were co-cultured at a 1:1 ratio. Consistent with the findings in parental Fcwf-4 cells, split luciferase activity increased in proportion to the cell fusion activity of the S protein. Among these variants, the S-GFP protein exhibited the strongest activity, followed by the PA-tagged S protein. In contrast, the untagged S protein sparsely induced syncytial formation in the culture wells (Fig. 6).

### The SARS-CoV-2 Spike protein induced syncytia

**Fig. 7:**
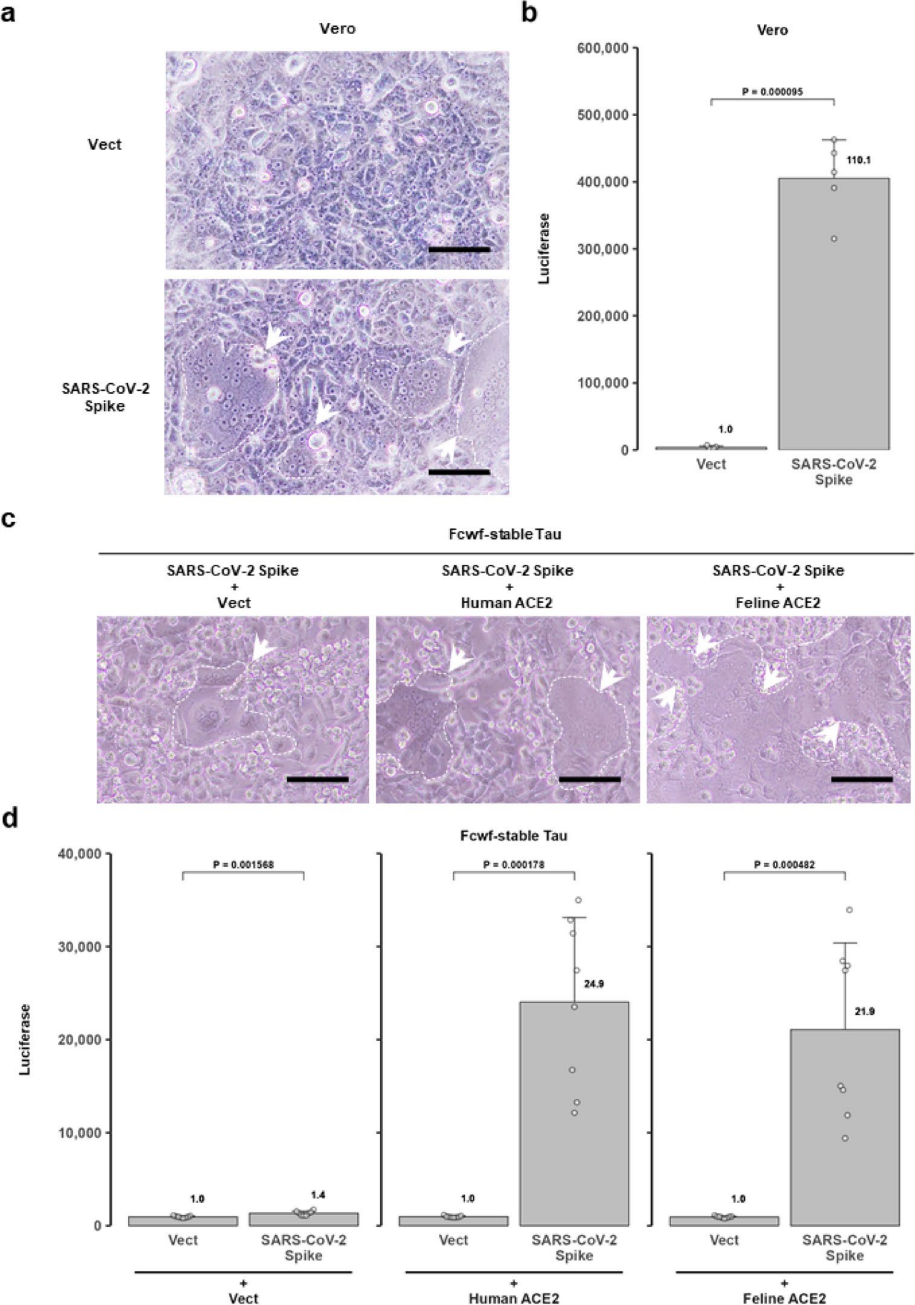
SARS-CoV-2 spike protein-induced cell fusion. (**a**) Visualization of SARS-CoV-2 spike expression-induced syncytia in parental Vero cells. Syncytia are indicated by a dashed line in light microscopy images captured 24 hpt. Scale bar: 100 μm. (**b**) Upregulation of split-Gluc expression in parental Vero cell culture media. Split-Gluc activity was determined at 24 hpt in co-cultured cells transiently expressing SARS-CoV-2 S and ssTau-Gn or ssTau-Gc. (**c**) Enhancement of SARS-CoV-2 spike-mediated syncytial formation by human and feline ACE2 in Fcwf-4-stable Tau cells. Syncytia are indicated by a dashed line in light microscopy images obtained 24 hpt. Scale bar: 100 μm. (**d**) ssTau-split Gluc activity of the Fcwf-stable Tau cells shown in panel (**c**). The empty control (Vect) or SARS-CoV-2 S expression vectors were co-transfected with human or feline ACE2 expression vectors. Split-Gluc activity was determined at 24 hpt.

SARS-CoV-2 induces cell‒cell fusion through its spike protein^22,23^. The expression of the SARS-CoV-2 S protein in Vero cells induced cell fusion and significantly increased ssTau-split Gluc activity in the culture medium (Fig. 7a,b).

SARS-CoV-2 infection has been reported in several non-human animal species, including domestic cats (*Felis catus*), with documented cases of mortality^23^. Therefore, Fcwf-stable Tau cells were transfected with the SARS-CoV-2 S vector in combination with either human or feline angiotensin-converting enzyme 2 (ACE2) vectors to investigate the susceptibility of feline cells to the SARS-CoV-2 S protein. The spike protein alone induced a relatively low level of cell‒cell fusion (1.4-fold greater than that of the control) (Fig. 7c and d). In contrast, co-expression of human ACE2, the primary receptor for the SARS-CoV-2 spike protein, significantly enhanced syncytial formation. Similarly, feline ACE2 enhanced cell fusion to a level comparable to human ACE2. This observation highlights the crucial role of human and feline ACE2 proteins in the mediation of SARS-CoV-2-induced cell fusion.

## Discussion

Although high-throughput quantitative analysis of cell fusion remains challenging, it is essential for screening compounds that inhibit viral infection and proliferation, as well as for the pathophysiological analysis of viral diseases. Traditional methods are less quantitative, have a lower throughput, and are more labor intensive. Fusion between multiple syncytia and detachment of fused cells frequently occurred in our experiments, particularly at high viral loads. Thus, microscopic methods that accurately measure the number and size of fused cells would be challenging in such experimental conditions. The measurement of luciferase activity is more suitable for high-throughput analysis. Therefore, we aimed to develop a luciferase-based method for the efficient and highly sensitive quantification of syncytia, a key downstream consequence of coronavirus entry into host cells via spike protein expression.

Traditional methods require cell lysis to release intracellular luciferase into the lysis buffer before measurement; this procedure is not feasible for large-scale screening or experiments in 384-well plates. A similar challenge has been observed in 96-well plates. When coronavirus-infected cells form syncytia containing intracellular luciferase, they are discarded with the culture media during the cell lysis step. This consequently hinders the measurement of luciferase, especially when a significant proportion of the cells have detached due to viral infection.

Our assay offers three key advantages in the measurement procedure. First, cell lysis is not required because the Tau-split Gluc protein is secreted into culture media by an ss sequence at the N-terminus of the Tau-split Gluc fusion protein, as shown in the present study. Second, a low volume of culture supernatant is sufficient for analysis. Finally, the processing of sample media, such as centrifugation, is not required before measurement. These advantages considerably reduce the analysis time. Our subsequent experiments, involving varying cell densities and supernatant dilutions, provided valuable insights into the sensitivity and dynamic range of this novel assay.

To evaluate the performance of our assay, syncytial cells were induced via FCoV infection and overexpression of spike protein in feline cell lines. Our results clearly demonstrate the restoration of split-Gluc enzymatic activity by cell‒cell fusion, which is consistent with our objective in developing this method. Although cells expressing ssTau-Gn and ssTau-Gc were mixed at a 1:1 ratio in the present study, ratios between 7:3 and 3:7 also yielded detectable signals, indicating the robustness of our assay. Moreover, we confirmed that the luciferase detected in the medium was secreted from the cells into the culture medium as a consequence of cell fusion. This result indicates that the assay can be performed independent of cell disruption or detachment from the culture plate. The linearity of luciferase activity in diluted media also confirmed the broad dynamic range of the ssTau-split Gluc assay. Furthermore, our assay demonstrated its applicability in the 384-well plate, where experiments using 50 µL of medium/well exhibited a higher signal-to-background ratio than those using 100 µL of medium in a 96-well plate, potentially enabling more efficient use of screening compounds. Although preliminary, our study showed that luciferase activity could be measured by directly dispensing coelenterazine into 384-well plates containing cultured cells. Our results indicate that this method has the potential for high-throughput screening applications. However, detailed experimental conditions will need to be optimized for each cell type.

Some fusion-detecting luciferase assays require transactivation of an intact luciferase gene upon cell‒cell fusion^24–26^. In this study, cells cultured for 48 h after transfection with the ssTau-split Gluc vector were used for FCoV infection experiments. A significant increase in luciferase activity in the culture media was observed 24 h post-infection. However, further investigation is required to clarify the differences in the time course necessary for a detectable increase in luciferase within the culture supernatant, as well as the impact of different experimental settings. Specifically, the shortest yet sufficient time for reliable luciferase detection warrants further determination.

The enhanced restoration of split Gluc activity upon FCoV spike protein overexpression suggests that our assay is valuable for investigating the cell fusion activity of this protein and potentially that of other coronavirus spike proteins. Moreover, overexpression of the GFP-tagged FCoV spike protein significantly enhanced cell fusion in this study. Although the precise mechanism underlying this enhancement remains unclear, we hypothesized that the GFP tag alters the conformation or expression level of the S protein^27^, thereby affecting its ability to induce cell‒cell fusion. Therefore, the ssTau-split Gluc system holds significant potential in the study of spike protein variants and the development of therapies targeting spike-mediated cell fusion.

Another advantage of our assay is that the inhibition of split Gluc restoration correlates with the suppression of cell fusion induced by infectious FCoV. This feature is relevant to anti-coronavirus drug screening. GS-441524, a nucleoside analog, significantly inhibited FCoV proliferation at concentrations as low as 1 µM. This result was quantitatively obtained using our ssTau-split Gluc system without excessively laborious experimental procedures, offering several advantages over traditional methods, including high-throughput capacity and reduced technical complexity. Our results also correlated with those obtained via the WST-8 assay, confirming that syncytial quantification accurately reflects cell survival. The successful quantification of the inhibitory effect of GS-441524 on FCoV proliferation using our assay highlights its potential as a valuable tool in the antiviral drug screening pipeline, particularly in the identification of compounds that suppress viral proliferation by directly interfering with the viral replication cycle after cell entry.

Tang et al.^28^ evaluated the effectiveness of fusion inhibitors against the fusion protein of respiratory syncytial virus using a luciferase reporter system. Although the present study did not demonstrate the applicability of our system for evaluating compounds that directly inhibit the fusion activity of the spike protein, we have initiated screening of compounds that suppress ssTau-split-Gluc restoration in FCoV S-GFP-mediated cell fusion. Several candidate compounds have been identified, and their potential to inhibit infectious FCoV is under investigation. The precise mechanism underlying Tau multimerization remains incompletely elucidated^29^, raising the possibility that certain compounds could inadvertently affect luciferase activity by interfering with this process, rather than directly inhibiting viral fusion or proliferation. Therefore, it is important to confirm whether the effect of a candidate compound is due to inhibition of viral processes and not an artifact of Tau multimerization interference. This could be addressed by first using control experiments with uninfected cells co-transfected with ssTau-Gn and ssTau-Gc, and then confirmed in infected cells by demonstrating a decrease in viral genome, protein, or titer.

One of the biggest limitations of the present study is that we used only FCoV as the infectious virus. Human herpes simplex virus-1 (HSV-1) can induce syncytium formation. A fusion-induced reporter gene expression system has successfully quantified HSV-induced syncytial cell formation^30^. Although HSV-1 induced Tau oligomerization^31^, it remains unclear whether HSV-induced Tau oligomerization restores split Gluc activity. Therefore, future studies are required to confirm whether our method is applicable to herpesviruses and other viral species in humans and animals.

Although antiviral drugs are required in the emergence of novel infectious viruses, studies using these viruses are restricted because of the risk of laboratory transmission and leakage from laboratories. Therefore, antiviral drugs should be safely screened using only the viral membrane fusion proteins for viruses that induce cell fusion. Our ssTau-Gluc assay is suitable for this purpose. Thus, we employed this assay to study the spike protein of SARS-CoV-2 to validate its applicability. Our results confirmed that the spike protein alone induced syncytial formation in feline cells, albeit with low efficiency. Co-expression with human ACE2 significantly enhanced syncytial formation, highlighting the critical role of this receptor in spike-mediated cell fusion. In addition, co-expression of feline ACE2 substantially increased syncytial formation via the SARS-CoV-2 spike protein in feline Fcwf-4 cells, confirming that feline ACE2 can mediate SARS-CoV-2 infection in cats. These findings demonstrate the susceptibility of cats to SARS-CoV-2 infection and confirm that our assay is effective for the functional analysis of coronavirus spike proteins. The successful application of our assay in the study of SARS-CoV-2 spike proteins and identification of feline ACE2 as an efficient mediator of fusion highlight the potential of this system for investigating host range and screening broad-spectrum antivirals targeting conserved fusion mechanisms across different coronaviruses.

Ultimately, our method has the potential for application in both primary screening using coronavirus spike proteins to identify candidate compounds, and in secondary screening using infectious coronaviruses to identify candidate compounds with industrialization potential. However, our system has certain limitations that warrant further consideration for broader applicability.

## Limitations

Our novel split luciferase complementation assay successfully quantified the cell-fusion caused by coronavirus spike proteins with high-throughput potency. However, the present study has some limitations in the interpretation of luciferase activity, and in the experimental design and protocol.

First, although our method can quantify fusion even when syncytia fuse with each other and detach from culture wells, this assay does not provide absolute values for the number and size of the syncytia. The number of syncytia indicates the infectious virus dose or the number of cells expressing spike proteins, whereas their size reflects the proliferative ability of the virus and expression level or fusogenic ability of the spike protein. Thus, experiments must be carefully interpreted using various infectious viral strains to determine the aspect of virus proliferation suppressed by candidate antiviral compounds. It is also necessary to confirm that the test compounds do not suppress Tau multimerization, which would lead to lower luciferase activity. This is especially important because the precise mechanism underlying Tau protein multimerization remains unclear^29^. As a complementary approach, a dual split protein (DSP) assay using a fusion protein of split luciferase and split GFP may be superior to the present method because it enables simultaneous high-throughput quantification via luciferase activity and size estimation of individual syncytia through GFP fluorescence, as demonstrated in SARS-CoV-2-induced syncytia^32^.

Second, luciferase assays using the traditional Gluc substrate coelenterazine are not ideal for extended time-course analyses. A DSP assay using split-*Renilla* luciferase and split-GFP, combined with a stable *Renilla* luciferase substrate such as EnduRen (Promega, Madison, WI, USA), may be a better solution for real-time quantification of luciferase activity over extended periods^32–34^. Because EnduRen is specifically for *Renilla* luciferase, we would need to investigate whether split-*Renilla* luciferase protein fragments fused with Tau protein can restore their enzymatic activity upon Tau protein multimerization.

Third, and most importantly, the present study only utilized infectious FCoV and the spike proteins of FCoV and SARS-CoV-2. Therefore, it remains unclear whether the ssTau-split Gluc system is applicable to cell fusion induced by other viruses. This aspect should be investigated using other infectious viruses, such as herpesviruses and retroviruses.

## Conclusion

The ssTau-split Gluc system provides a robust and versatile platform for high-throughput screening of antiviral drugs. It is particularly valuable for studying the fusogenic activity of numerous coronavirus spike protein variants, thereby eliminating the biosafety concerns associated with handling live, pathogenic viruses in early drug screening stages. Despite its limitations, our novel assay provides valuable information reflecting the overall formation of coronavirus-induced syncytia within the entire well by measuring luciferase activity secreted into the culture media. These findings contribute to research integrating viral pathogenicity analysis and antiviral drug development by enabling high-throughput screening. Our findings provide a theoretical basis for the identification of novel antiviral candidates, as demonstrated by our initial screening efforts targeting FCoV S-mediated cell fusion. Although future studies are required to address the identified limitations, our novel assay can be a valuable tool in drug discovery pipelines and in the study of the fusion-inducing ability of coronavirus spike proteins.

## Methods

### Cells and viruses

CRFK, Fcwf-4, and Vero cells were cultured in Dulbecco’s modified Eagle’s medium (Nacalai Tesque, Kyoto, Japan) supplemented with 10% fetal bovine serum, 4.5 g/L glucose, penicillin (100 U/mL), and streptomycin (100 µg/mL). The serotype II FCoV strain M91-267^35^ was used for viral infection.

### Expression vectors of the S genes of FCoV and SARS-CoV-2, as well as human and feline ACE2

To construct an expression vector for the S gene of FCoV strain M91-267, viral RNA was extracted from the FCoV strain M91-267 viral stock using the QIAamp Viral RNA Kit (QIAGEN, Hilden, Germany) and reverse-transcribed into complementary DNA (cDNA) using the PrimeScript 1st Strand cDNA Synthesis Kit (Takara Bio, Shiga, Japan). The S gene encoding the spike protein was amplified from cDNA through polymerase chain reaction using a high-fidelity DNA polymerase KOD One PCR Master Mix -Blue- (TOYOBO, Osaka, Japan). The amplicons were subsequently cloned and inserted into a pCI mammalian expression vector (Promega) using an In-Fusion HD Cloning Kit (Takara Bio). The expression vectors containing the amplicon were subsequently transduced into a chemically competent DH5α *ECOS* competent *E. coli* strain (NIPPON GENE, Tokyo, Japan) to generate a wild type with or without a 3′ terminal PA sequence tag, or a green fluorescent protein variant of the AcGFP1 sequence-tagged M91-267 S gene expression vector.

The S genes of the SARS-CoV-2 isolate Wuhan-Hu-1 (QHD43416)^36^ and human ACE2 (NP_068576.1) were amplified via overlap extension polymerase chain reaction using codon-optimized primers. A feline ACE2 sequence (NP_001034545) was amplified from the clinical sample of a cat. Next, the sequences to be expressed in cells were cloned and inserted into a pCI vector. Miniprep and midiprep were performed using a QIAprep Spin Miniprep Kit (QIAGEN) and NucleoBond Xtra Midi Kit (Takara Bio), respectively. The sequences of these expression vectors are shown in Supplementary Fig. 4.

### Secretory forms of Tau-split gluc expression vectors

For the split Gluc assay, the human *MAPT* gene encoding the full-length Tau protein isoform 2 (Tau 2N4R, 441 aa, NP_005901) was cloned and inserted into a pCI vector containing an ss sequence of the human immunoglobulin κ light chain. The split Gluc fragments (Gn for aa 2–93 and Gc for aa 94–169) were appended to the C-terminus of a Tau protein with a flexible linker to generate the pCI-ssTau-Gn and pCI-ssTau-Gc plasmids, respectively (Fig. 1a). The ssTau-split Gluc sequences were also cloned and inserted into a pIRESneo3 vector (Clontech, Mountain View, CA, USA) to establish Fcwf-4 cells that stably and independently expressed either the ssTau-Gn or ssTau-Gc protein (Fcwf-stable Tau cells). The ORF sequences of these expression vectors are shown in Supplementary Fig. 4.

### Cell transfection and co-culture

The pCI-ssTau-split Gluc vectors were independently introduced into the Fcwf-4 and CRFK cells to detect infectious FCoV-induced cell fusion. Cells were transfected with the respective plasmids using Lipofectamine LTX with PLUS Reagent (Thermo Fisher Scientific, Waltham, MA, USA). The transfected cells were cultured for 48 h and retrieved through trypsinization. The obtained cells were resuspended in a cryopreservation reagent CELLBANKER 1 (Takara Bio) and stored at – 80 °C until use. This process is practical because a batch of identical transfected cells could be prepared for multiple experiments, thereby minimizing the difference among the lots. To perform experiments, the cells were thawed, washed, and then co-cultured with or without FCoV at a density of 3–5 × 10^4^/100 µL/well (3–8 wells/group) in 96-well plates. These cells were cultured at 37 °C for up to 24 h. In the experiment using a 384-well plate, cells were cultured with or without FCoV at a density of 5–0.16 × 10^4^ cells in an indicated volume of medium in a well and incubated at 37 °C for 24 h.

For transient expression of spike proteins, S genes of FCoV or SARS-CoV-2 vectors were introduced into indicated cells, along with the ssTau-Gn or ssTau-Gc vector at the ratio of 10:1 in micrograms. The cells were cultured at 37 °C for 2 h, trypsinized, washed, and frozen once before co-culture.

### Establishment of Fcwf-4 cells stably expressing ssTau-split gluc

Fcwf-4 cells were transfected with pIRESneo3-ssTau-split Gluc vectors and subsequently cultured in the presence of G418 sulfate. The obtained colonies were screened to determine the expression level of ssTau-split Gluc. Co-culture was performed at a density of 3 × 10^4^ cells/100 µL/well (8 wells/group) in a 96-well plate (Gn: Gc = 1:1 ratio).

### Determination of split gluc activity

Twenty microliters of culture medium was transferred directly to a 96-well white-colored microplate without any processing, such as centrifugation, and mixed with 50 µL of coelenterazine buffer (100 mM Tris-HCl, pH 7.5; 10 mM sodium sulfite; 1.3 µM native coelenterazine (Biotium, Fremont, CA, USA)) to measure Gluc activity. Luminescence was quantified with a NIVO Multimode Microplate Reader equipped using an automatic coelenterazine buffer dispenser (PerkinElmer, Waltham, MA, USA). In the experiments shown in Supplementary Fig. 2b, coelenterazine buffer was injected directly into white 384-well plates designed for adherent cell culture, in which Fcwf-4 cells had been cultured.

### Time-lapse analysis

Fcwf-4 cells were transfected with GFP-tagged FCoV-S and cultured for 24 h in a time-lapse imaging system using a BZ-X800 microscope (Keyence, Osaka, Japan). Photographs were captured at 5-min intervals.

### Nucleoside analog treatment

GS-441524 (R&D Systems, Minneapolis, MN, USA) was dissolved in DMSO to obtain a stock concentration of 100 mM. The drugs were diluted to the desired final concentration in a culture medium. Fcwf-4 cells transiently expressing ssTau-split Gluc were simultaneously treated with GS-441524 and infected with FCoV. To this end, the cells were co-cultured at a density of 5 × 10^4^ cells/well (6 wells per group) in a 96-well plate. They were simultaneously treated with varying doses of GS-441524 and infected with FCoV (1,000 TCID_50_). Split Gluc activity in 20 µL medium was determined at 24 h post-infection. Next, 10 µL of Cell Counting Kit-8 (Dojindo, Kumamoto, Japan) solution was added to each well and incubated for 1 h at 37 °C. Finally, absorbance was measured at OD_450 nm using the NIVO multimode microplate reader.

### Statistical analysis

Differences in the mean luciferase values between the two groups were analyzed through Welch’s two-tailed t-test using R statistical package (version 4.4.3)^37^ and RStudio (version 024.12.0.467)^38^. *P <* 0.05 was considered statistically significant. The maximal information coefficient^20^ was calculated using the *minerva* package.

## Electronic supplementary material

Below is the link to the electronic supplementary material.

Supplementary Material 1

Supplementary Material 2

Supplementary Material 3

## Data Availability

The datasets generated and analyzed during the current study are available upon request from the corresponding author.
