# Supplementary figures and images for: A novel luciferase-based assay for quantifying coronavirus-induced syncytia
Source: Sci Rep. 2025 May 20;15:17423. doi: 10.1038/s41598-025-02037-4 (PMC12092680; doi:10.1038/s41598-025-02037-4)

## Supplementary Figure 1

**a**

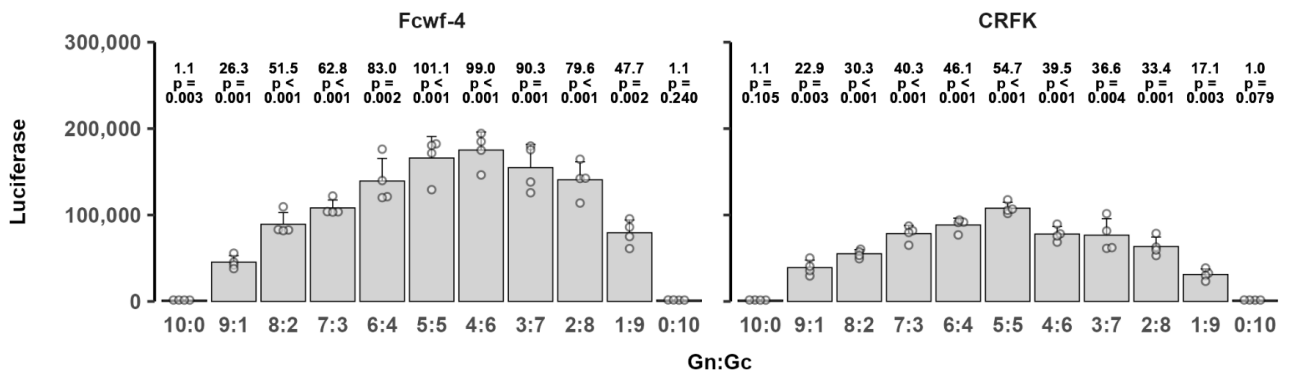**b**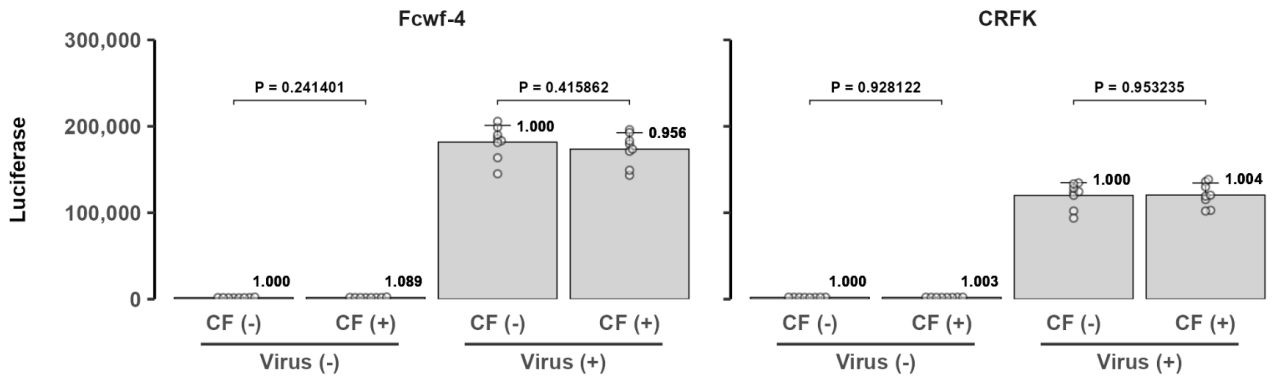

**C**

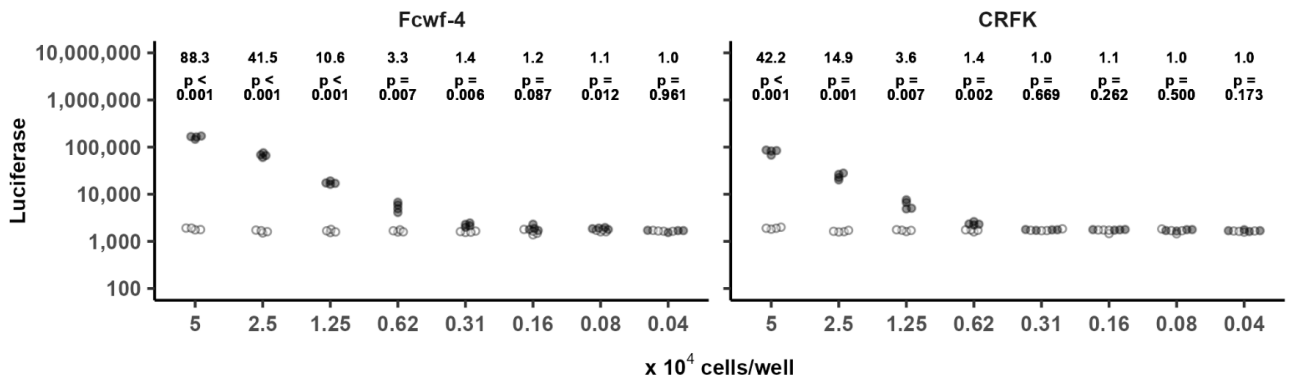

**d**

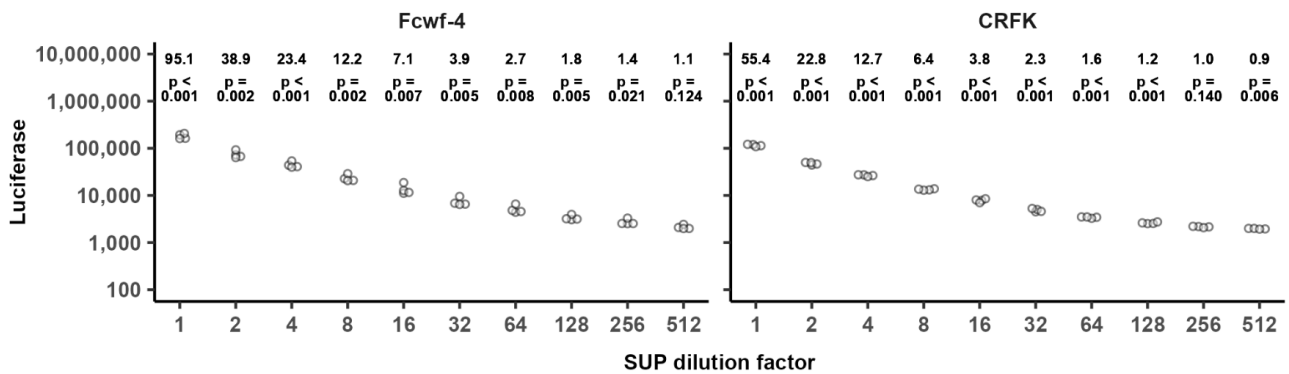

Supplementary Figure 2

a

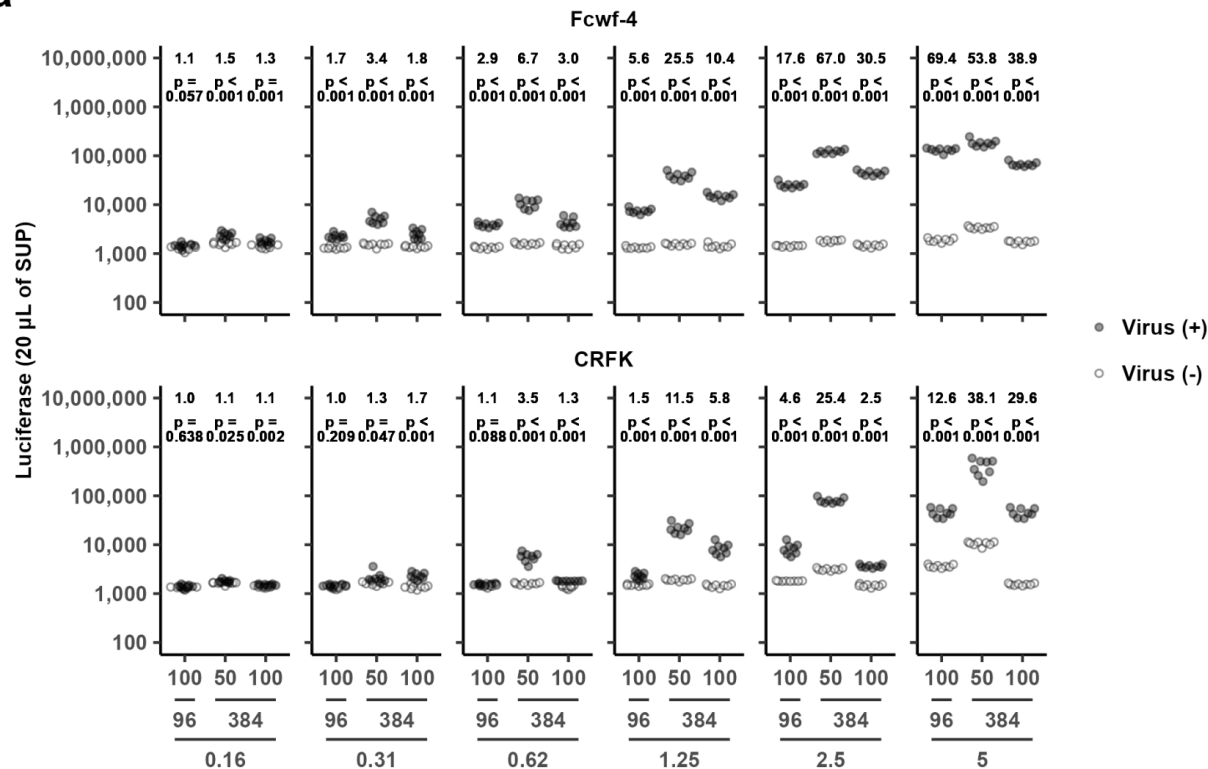

b

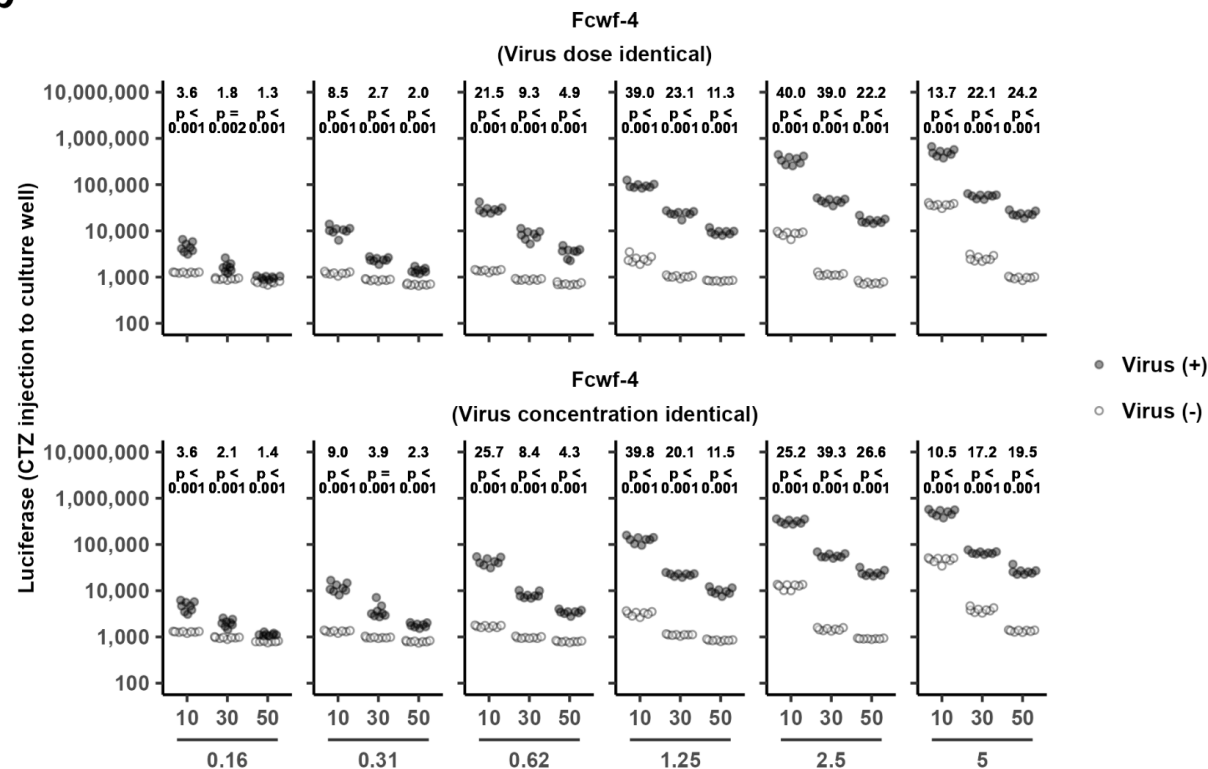

Supplementary Figure 3

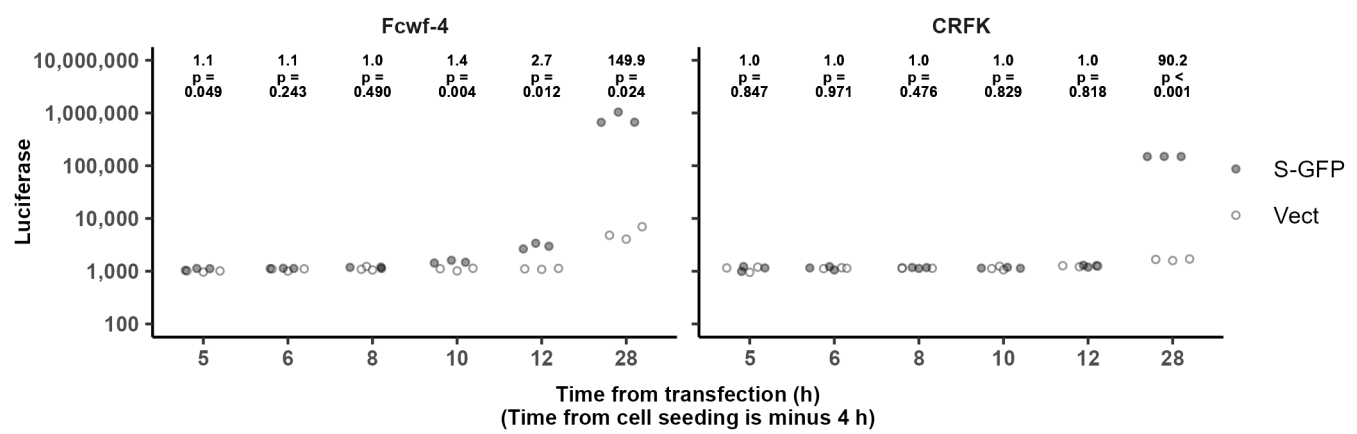

Supplement: Supplementary file 2 — Supplementary Material 2 [file 41598_2025_2037_MOESM2_ESM.pdf]
