# Supplementary material for: A novel luciferase-based assay for quantifying coronavirus-induced syncytia
Source: Sci Rep. 2025 May 20;15:17423. doi: 10.1038/s41598-025-02037-4 (PMC12092680; doi:10.1038/s41598-025-02037-4)
Supplement: Supplementary file 3 — Supplementary Material 3 [file 41598_2025_2037_MOESM3_ESM.docx]

Supplementary Fig. 1. Sensitivity of ssTau-split Gluc assay.

1. Optimal ratio to co-culture cells expressing either ssTau-Gn or ssTau-Gc. Cells expressing ssTau-Gn were co-cultured with that expressing ssTau-Gc at the ratio of 10:0 to 0:10. Value of luciferase activity relative to non-infected control (not shown) at each ratio were shown at the top of bar. Data were expressed as mean ± SD with raw values as circles.
2. Secretion of ssTau-Gluc into culture media. The luciferase activity was measured in culture supernatants that were and wer not subjected to centrifuge at 10,000 x g, 1 min before measurement of luciferase activity. Fold-increase and p-values on the top of bars were calculated based on comparisons without and with centrifugation for both uninfected (not shown) and FCoV-infected cells, respectively. Data were expressed as mean ± SD with raw values as circles.
3. Relationship between density of cells infected with FCoV in a 96-well plate and luciferase activity in the culture media. Closed (●) and open (○) circles indicate raw luciferase values of FCoV-infected and non-infected cells, respectively. Fold-increase and p-values in the comparison with non-infected cells at each cell density were indicated.
4. Dilutional linearity of luciferase activity. Culture supernatant samples of cells infected with FCoV were subjected to two-fold serial dilution using 10% FBS-DMEM before measurement. Fold-increase and p-values in the comparison with non-diluted samples of non-infected cells (not shown) were indicated above and below the circles showing raw data, respectively.

Supplementary Fig. 2. Applicability of ssTau-split Gluc assay to 384-well format.

1. Luciferase of Fcwf-4 (top) and CRFK (bottom) cells in 50 μL (“50”) or 100 μL (“100”) of medium in 96- (“96”) and 384-(“384”) well plate. 0.16, 0.31, 0.62, 1.25, 2.5, and 5 indicate cell density in “× 10^4^ cells/well”. Twenty microliters of SUP was used to determine luciferase activity. Closed and open circles (● and ○) indicate the raw luciferase values of FCoV-infected and non-infected wells, respectively.
2. Luciferase of Fcwf-4 cells in 10, 30, and 50 μL (“10”, “30”, and “50”) of medium in a 384-well plate. 0.16 ~ 5 indicate cell density in “× 10^4^ cells/well”. Luciferase activity was measured by directly dispensing 50 μL of coelenterazine (CTZ) buffer into wells of culture plates. In the top panel, the virus dose is maintained constant across all cell densities, independent of the culture medium volume. In the bottom panel, the virus concentration is kept constant. Closed and open circles (● and ○) indicate the raw luciferase values of FCoV-infected and non-infected wells, respectively.

Supplementary Fig. 3. Time-course analysis of luciferase expression.

Fcwf-4 and CRFK cells transfected with FCoV S-GFP (●) or empty vector (Vect, ○) with either ssTau-Gn or ssTau-Gc were co-cultured at 1:1 ratio. Luciferase in culture media was determined at the indicated times. Since there was a 4-hour interval between gene transfection and cell seeding, the first hour of culture was equivalent to 5 hours post-transfection. Consequently, the displayed "Time from transfection" range of 5–28 h corresponds to a "Time from cell seeding to a culture plate" range of 1–24 h, respectively. Fold-increase and p-values in the comparison with non-infected cells at each time point were indicated.

Supplementary Fig. 4. ORF of FCoV and SARS-CoV-2 S genes, human and feline ACE2 in the pCI mammalian expression vector.

Supplementary Video S1. Time-lapse images of Fcwf-4 cells expressing FCoV S-GFP protein. A syncytial cell is formed in a middle-left area of the video. Scale bar: 100 μm.

**Supplementary Fig. 4**

1. ssTau-Gn (TGn):Secretory signal and linker sequences are underscored with a straight and wavy line, respectively.

ATGGACATGAGAGTGCCTGCCCAGCTGCTGGGACTGCTGCTGCTGTGGCTGAGAGGCGCCAGATGTATGGCCGAACCTAGACAAGAGTTCGAAGTGATGGAAGATCACGCCGGCACATACGGCCTGGGCGACAGAAAAGATCAAGGCGGCTACACCATGCACCAGGACCAAGAGGGCGATACAGACGCCGGCCTGAAAGAGTCTCCTCTGCAGACCCCTACAGAGGACGGCTCTGAGGAACCTGGCAGCGAGACATCTGACGCCAAGAGCACACCTACCGCCGAGGATGTTACAGCCCCTCTGGTTGATGAAGGCGCCCCTGGAAAACAGGCTGCCGCTCAGCCTCACACAGAGATCCCTGAGGGAACCACAGCCGAGGAAGCCGGCATTGGCGATACACCCAGCCTGGAAGATGAAGCCGCCGGACATGTGACCCAGGCCAGAATGGTGTCCAAGAGCAAGGATGGCACCGGCAGCGACGACAAGAAAGCCAAAGGCGCCGATGGCAAGACCAAGATCGCTACCCCTAGAGGCGCTGCTCCTCCTGGACAGAAAGGACAGGCCAACGCCACAAGAATCCCCGCCAAAACACCTCCTGCTCCTAAGACACCTCCTAGCAGCGGAGAGCCTCCTAAGAGCGGCGATAGAAGCGGCTATAGCAGCCCTGGCTCTCCTGGCACACCTGGCTCTAGAAGCAGAACCCCTAGCCTGCCTACACCTCCAACCAGAGAACCCAAGAAAGTGGCCGTCGTGCGGACCCCTCCTAAGTCTCCATCTAGCGCCAAGTCCAGACTGCAGACAGCCCCTGTGCCTATGCCTGACCTGAAGAACGTGAAGTCCAAGATCGGCAGCACCGAGAACCTGAAACACCAGCCTGGCGGAGGCAAGGTGCAGATCATCAACAAGAAGCTGGACCTGAGCAACGTGCAGAGCAAGTGCGGCAGCAAGGACAACATCAAGCACGTGCCAGGCGGCGGATCTGTGCAGATCGTGTACAAGCCTGTGGACCTGTCCAAAGTGACCAGCAAGTGTGGCTCCCTGGGCAACATCCATCACAAACCAGGCGGAGGACAGGTGGAAGTGAAGTCCGAGAAACTGGACTTCAAGGACCGGGTGCAGTCTAAGATCGGAAGCCTGGACAATATCACTCACGTTCCCGGCGGAGGAAACAAGAAGATCGAGACACACAAGCTGACCTTCCGCGAGAACGCCAAGGCCAAGACAGATCACGGCGCCGAGATTGTGTATAAGAGCCCTGTGGTGTCCGGCGACACAAGCCCTAGACACCTGTCTAACGTGTCCAGCACCGGCTCCATCGACATGGTGGATTCTCCACAGCTGGCCACACTGGCCGATGAAGTGTCTGCCTCTCTGGCCAAGCAGGGCCTGGGCGGAGGCGGATCTGGGGGAGGGGGATCTAAGCCCACCGAGAACAACGAGGACTTCAATATCGTGGCCGTGGCCAGCAACTTCGCCACCACCGATCTGGACGCCGACAGAGGCAAGCTGCCCGGCAAGAAACTGCCCCTGGAAGTGCTGAAAGAGATGGAAGCCAACGCCCGGAAGGCCGGCTGTACCAGAGGCTGCCTGATCTGCCTGAGCCACATCAAGTGCACCCCCAAGATGAAGAAGTTCATCCCCGGCAGATGCCACACCTACGAGGGCGACAAAGAGTCTGCCCAGGGCGGCATCGGATGA

1. ssTau-Gc (TGc) :Secretory signal and linker sequences are underscored with a straight and wavy line, respectively.

ATGGACATGAGAGTGCCTGCCCAGCTGCTGGGACTGCTGCTGCTGTGGCTGAGAGGCGCCAGATGTATGGCCGAACCTAGACAAGAGTTCGAAGTGATGGAAGATCACGCCGGCACATACGGCCTGGGCGACAGAAAAGATCAAGGCGGCTACACCATGCACCAGGACCAAGAGGGCGATACAGACGCCGGCCTGAAAGAGTCTCCTCTGCAGACCCCTACAGAGGACGGCTCTGAGGAACCTGGCAGCGAGACATCTGACGCCAAGAGCACACCTACCGCCGAGGATGTTACAGCCCCTCTGGTTGATGAAGGCGCCCCTGGAAAACAGGCTGCCGCTCAGCCTCACACAGAGATCCCTGAGGGAACCACAGCCGAGGAAGCCGGCATTGGCGATACACCCAGCCTGGAAGATGAAGCCGCCGGACATGTGACCCAGGCCAGAATGGTGTCCAAGAGCAAGGATGGCACCGGCAGCGACGACAAGAAAGCCAAAGGCGCCGATGGCAAGACCAAGATCGCTACCCCTAGAGGCGCTGCTCCTCCTGGACAGAAAGGACAGGCCAACGCCACAAGAATCCCCGCCAAAACACCTCCTGCTCCTAAGACACCTCCTAGCAGCGGAGAGCCTCCTAAGAGCGGCGATAGAAGCGGCTATAGCAGCCCTGGCTCTCCTGGCACACCTGGCTCTAGAAGCAGAACCCCTAGCCTGCCTACACCTCCAACCAGAGAACCCAAGAAAGTGGCCGTCGTGCGGACCCCTCCTAAGTCTCCATCTAGCGCCAAGTCCAGACTGCAGACAGCCCCTGTGCCTATGCCTGACCTGAAGAACGTGAAGTCCAAGATCGGCAGCACCGAGAACCTGAAACACCAGCCTGGCGGAGGCAAGGTGCAGATCATCAACAAGAAGCTGGACCTGAGCAACGTGCAGAGCAAGTGCGGCAGCAAGGACAACATCAAGCACGTGCCAGGCGGCGGATCTGTGCAGATCGTGTACAAGCCTGTGGACCTGTCCAAAGTGACCAGCAAGTGTGGCTCCCTGGGCAACATCCATCACAAACCAGGCGGAGGACAGGTGGAAGTGAAGTCCGAGAAACTGGACTTCAAGGACCGGGTGCAGTCTAAGATCGGAAGCCTGGACAATATCACTCACGTTCCCGGCGGAGGAAACAAGAAGATCGAGACACACAAGCTGACCTTCCGCGAGAACGCCAAGGCCAAGACAGATCACGGCGCCGAGATTGTGTATAAGAGCCCTGTGGTGTCCGGCGACACAAGCCCTAGACACCTGTCTAACGTGTCCAGCACCGGCTCCATCGACATGGTGGATTCTCCACAGCTGGCCACACTGGCCGATGAAGTGTCTGCCTCTCTGGCCAAGCAGGGCCTGGGCGGAGGCGGATCTGGGGGAGGGGGATCTGAAGCCATCGTGGACATCCCCGAGATCCCCGGCTTCAAGGACCTGGAACCCATGGAACAGTTTATCGCCCAGGTGGACCTGTGCGTGGACTGCACCACAGGCTGTCTGAAGGGCCTGGCCAACGTGCAGTGCAGCGACCTGCTGAAGAAGTGGCTGCCCCAGAGATGCGCCACCTTCGCCTCTAAGATCCAGGGACAGGTGGACAAGATCAAGGGCGCTGGCGGCGACTGA

1. FCoV strain M91-267 S gene CDS

ATGATTGTGCTCGTAACTTGCATTTTATTGTTATGCTCATACCACATTGCTTCGAGTACGTCGAATAATGATTGTAGACAAGTTAACGTAACACAATTAGATGGCAATGAAAACCTTATTAAAGACTTTTTGTTTCAAAACTTTAAAGAAGAAGGAAGTGTAGTTGTTGGTGGTTACTACCCGACAGAGGTGTGGTATAACTGTTCTAGGACAGCAACAACTACTGCCTATGAGTATTTCAGTAACATACATGCTTTCTATTTTGATATGGAAGCCATGGAAAATAGTACTGGTAATGCACGGGGTAAACCTTTATTATTCCATGTGCATGGTGAGCCGGTTAGTGTTATCATATATATATCGGCTTATACAAATGATGTGCAACAAAGGCCACTTTTAAAACATGGATTAGTGTGCATAATTAATAGTCGCAATATTGACTATAATAAATTCACCAGTAACCAGTGGGGTTCCATATGTACGGGTAATGACAGAAAAATCCCTTTTTCTGTCATACCCACGGATAATGGAACAAAAATCTACGGGCTTGAGTGGAATGATGAATTTGTTACAGCATACATTAGTGGCCGTTCTTATAATTGGAACATCAATAATAATTGGTTTAACAATGTCACACTTTTTTACTCTCGCTCAAGTACTGCCACATGGCAACACAGTGCTGCATATGTTTACCAAGGTGTTTCTAACTTCACTTATTACAAGTTAAATAACACCAATGGTCTAAAAACCTATGAATTATGTGAAGATTATGAATATTGCACTGGCCACGCCACTAATATCTTTGCCCCAACGGTGGGAGGTTACATACCTGATGGATTTAGTTTTAACAATTGGTTTTTGCTTACAAATAGCTCCACTTTTATTAGTGGCAGATTTGTGACAAATCAGCCATTGTTAGTTAATTGCTTGTGGCCAGTGCCTAGTTTTGGTGTTGCAGCACAAGAATTTTGTTTTGAAGGTGCCCAGTTTAGCCAATGTAATGGTGTGTCTTTAAATAACACAGTGGATGTCATTAGATTCAACCTTAATTTTACTGCAGATGTACAATCTGGCATGGGTGCTACAGTATTTTCACTGAATACAACAGGTGGTGTCATTCTTGAGATTTCTTGTTATAATACAGTGAGCGAGTCAAGTTTTTACAGTTATGGTGAAATTCCATTCGGCGTAACTGATGGACCGCGTTACTGTTATGTACTCCATAATGGCACGGCTCTTAAGTATTTAGGAACATTGCCACCTAGTGTCAAGGAAATTGCTATTAGTAAGTGGGGCCATTTTTATATTAATGGTTACAATTTCTTTAGCACTTTTCCTATTGATTGTATATCTTTCAACTTAACCACTGGTGCTAGTGGAGCTTTCTGGACAATTGCTTACACATCGTACACTGAAGCATTAGTACAAGTTGAAAACACAGCTATTAAGAAGGTGACGTATTGTAACAGTCACATTAATAACATCAAATGTTCTCAACTTACTGCTAATTTGCAAAATGGATTTTATCCTGTTGCCTCAAGTGAAGTAGGTCTCGTTAATAAGAGTGTTGTGTTACTACCTATCTTTCTCACACATACCGCTGTCAATATAACGATTGATCTTGGTATGAAGCGTAGTGGCTACGGTCAACCCATAGCTTCATCATTAAGTAACATCACACTACCGATGCAGGATAATAACACCGATGTGTACTGCATTCGTTCTAACCAATTTTCAGTTTATGTCCACTCCACTTGCAAAAGTTCTTTATGGGACAATATTTTTGATTTAGATTGCACAGATGTTTTATATGCCACAGCTGTTATAAAAACTGGTACTTGCCCCTTCTCATTTGATAAATTGAATAATTACCTAACTTTTAACAAGTTTTGTTTGTCGTTGAGTCCTACCGGTGCCAACTGTAAGTTTGATGTTGCCGCCCGTACAAGAACCAATGAGCAGGTTGTTAGAAGTTTATATGTAATATATGAAGAAGGAGACAACATAGTGGGTGTACCGTCTGATAATAGTGGTCTTCACGATCTGTCAGTGTTACACTTAGACTCCTGTACAGATTACAATATATATGGTAGAACTGGTGTTGGTATTATTAGACAAACTAACAGCACAATACTTAGTGGCTTATATTACACCTCACTATCAGGTGATTTGTTAGGTTTTAAAAATGTTAGTGATGGTGTTGTCTATTCTGTGACACCATGTGATGTAAGCGCACAAGCGGCTGTTATTGATGGTGCCATAGTTGGAGCTATGACCTCCATTAATAGTGAACTGTTAGGTCTAACACATTGGACAACAACACCTAATTTTTATTACTACTCTATATATAATTACACAAATGAGAGAACTCGTGGCACTGCAATTGACAGTAACGATGTTGATTGTGAACCTATCATAACCTATTCTAACATAGGTGTTTGTAAAAATGGAGCATTGGTCTTTATTAACGTCACACATGCTGATGGAGACGTTCAACCAATTAGCACTGGCAATGTCACGATACCCACAAACTTTACCATATCTGTGCAAGTCGAATACATCCAGGTTTACACCACACCAGTGTCAATAGATTGTTCTAGATACGTTTGTAATGGTAACCCTAGATGTAATAAATTGTTAACACAATATGTCTCTGCATGTCAAACTATTGAGCAAGCGCTTGCAATGAGTGCCAGACTTGAAAACATGGAAGTTGATTCCATGTTGTTTGTTTCAGAAAATGCCCTTAAATTGGCATCTGTTGAGGCGTTCAATAGTACAGAACATTTAGATCCTATTTACAAAGAATGGCCTAACATAGGTGGTTCTTGGCTAGGAGGTCTAAAAGACATACTTCCGTCCCATAATAGCaaacgtaagtaccgtTCTGCTATAGAAGACTTGCTTTTTGATAAAGTCGTAACTTCTGGTCTAGGTACAGTTGATGAAGATTATAAACGTTGTACAGGTGGTTATGACATAGCTGACTTAGTTTGTGCACAATATTACAATGGCATCATGGTTCTACCTGGTGTGGCTAATGATGACAAGATGACTATGTACACAGCCTCTCTTGCAGGTGGTATAACATTAGGTGCACTAGGTGGTGGCGCCGTGGCTATACCTTTTGCAGTAGCAGTTCAGGCTAGACTTAATTATGTTGCTCTACAAACTGATGTATTGAATAAAAACCAGCAGATCCTGGCTACTGCTTTCAACCAAGCTATTGGTAACATTACACAGGCATTTGGTAAGGTTAATGATGCTATACATCAAACATCACAAGGTCTTGCCACTGTTGCTAAAGCCTTGGCAAAAGTGCAAGATGTTGTTAACACACAAGGGCAAGCTTTAAGCCACCTAACAGTACAACTGCAAAATAACTTTCAAGCCATTAGTAGTTCTATTAGTGACATTTATAATAGGCTTGATGAATTGAGTGCTGATGCACAAGTTGACAGGCTGATTACAGGAAGACTTACAGCACTTAATGCCTTTGTGTCTCAGACTTTAACCAGACAAGCAGAGGTTAGGGCTAGTAGACAACTTGCCAAAGACAAAGTTAATGAATGCGTTAGGTCTCAATCCCAGAGATTTGGATTCTGTGGTAATGGTACACATTTGTTTTCACTTGCAAATGCAGCACCAAATGGCATGATTTTCTTTCACTCAGTACTATTACCAACAGCTTATGAAACTGTGACAGCCTGGTCAGGTATTTGTGCGTCAGATGGCGATCGCACTTTTGGACTTGTAGTTAAGGATGTCCAGCTGACGCTATTCCGCAACCTAGATGACAAGTTTTATTTGACTCCCAGAACTATGTATCAGCCTAGAGCTGCAACTAGTTCTGATTTTGTTCAAATTGAGGGGTGCGATGTGTTGTTTGTCAATGCTACTGTAATTGAATTGCCTAGTATTATACCTGACTACATCGACATTAATCAGACTGTTCAAGACATATTAGAAAACTACAGACCAAACTGGACTGTACCTGAATTGACACTTGACATTTTTAACGCAACCTATTTAAATCTGACTGGTGAAATTGATGACTTAGAATTTAGGTCAGAAAAGCTACATAACACTACTGTAGAACTTGCCATTCTCATTGACAACATTAACAATACATTAGTCAATCTTGAATGGCTCAATAGAATTGAAACTTATGTGAAATGGCCTTGGTATGTGTGGCTACTAATAGGCTTAGTAGTAGTATTTTGCATACCGCTATTGCTATTTTGCTGTTGTAGTACAGGTTGCTGTGGATGCATAGGTTGTTTGGGAAGTTGTTGTCACTCTATTTGTAGTAGAAGACAATTTGAAAATTATGAACCAATTGAAAAAGTGCATGTCCACTAG

1. FCoV strain M91-267 S gene with PA tag (S-PA):PA tag sequence is indicated by lower case.

ATGATTGTGCTCGTAACTTGCATTTTATTGTTATGCTCATACCACATTGCTTCGAGTACGTCGAATAATGATTGTAGACAAGTTAACGTAACACAATTAGATGGCAATGAAAACCTTATTAAAGACTTTTTGTTTCAAAACTTTAAAGAAGAAGGAAGTGTAGTTGTTGGTGGTTACTACCCGACAGAGGTGTGGTATAACTGTTCTAGGACAGCAACAACTACTGCCTATGAGTATTTCAGTAACATACATGCTTTCTATTTTGATATGGAAGCCATGGAAAATAGTACTGGTAATGCACGGGGTAAACCTTTATTATTCCATGTGCATGGTGAGCCGGTTAGTGTTATCATATATATATCGGCTTATACAAATGATGTGCAACAAAGGCCACTTTTAAAACATGGATTAGTGTGCATAATTAATAGTCGCAATATTGACTATAATAAATTCACCAGTAACCAGTGGGGTTCCATATGTACGGGTAATGACAGAAAAATCCCTTTTTCTGTCATACCCACGGATAATGGAACAAAAATCTACGGGCTTGAGTGGAATGATGAATTTGTTACAGCATACATTAGTGGCCGTTCTTATAATTGGAACATCAATAATAATTGGTTTAACAATGTCACACTTTTTTACTCTCGCTCAAGTACTGCCACATGGCAACACAGTGCTGCATATGTTTACCAAGGTGTTTCTAACTTCACTTATTACAAGTTAAATAACACCAATGGTCTAAAAACCTATGAATTATGTGAAGATTATGAATATTGCACTGGCCACGCCACTAATATCTTTGCCCCAACGGTGGGAGGTTACATACCTGATGGATTTAGTTTTAACAATTGGTTTTTGCTTACAAATAGCTCCACTTTTATTAGTGGCAGATTTGTGACAAATCAGCCATTGTTAGTTAATTGCTTGTGGCCAGTGCCTAGTTTTGGTGTTGCAGCACAAGAATTTTGTTTTGAAGGTGCCCAGTTTAGCCAATGTAATGGTGTGTCTTTAAATAACACAGTGGATGTCATTAGATTCAACCTTAATTTTACTGCAGATGTACAATCTGGCATGGGTGCTACAGTATTTTCACTGAATACAACAGGTGGTGTCATTCTTGAGATTTCTTGTTATAATACAGTGAGCGAGTCAAGTTTTTACAGTTATGGTGAAATTCCATTCGGCGTAACTGATGGACCGCGTTACTGTTATGTACTCCATAATGGCACGGCTCTTAAGTATTTAGGAACATTGCCACCTAGTGTCAAGGAAATTGCTATTAGTAAGTGGGGCCATTTTTATATTAATGGTTACAATTTCTTTAGCACTTTTCCTATTGATTGTATATCTTTCAACTTAACCACTGGTGCTAGTGGAGCTTTCTGGACAATTGCTTACACATCGTACACTGAAGCATTAGTACAAGTTGAAAACACAGCTATTAAGAAGGTGACGTATTGTAACAGTCACATTAATAACATCAAATGTTCTCAACTTACTGCTAATTTGCAAAATGGATTTTATCCTGTTGCCTCAAGTGAAGTAGGTCTCGTTAATAAGAGTGTTGTGTTACTACCTATCTTTCTCACACATACCGCTGTCAATATAACGATTGATCTTGGTATGAAGCGTAGTGGCTACGGTCAACCCATAGCTTCATCATTAAGTAACATCACACTACCGATGCAGGATAATAACACCGATGTGTACTGCATTCGTTCTAACCAATTTTCAGTTTATGTCCACTCCACTTGCAAAAGTTCTTTATGGGACAATATTTTTGATTTAGATTGCACAGATGTTTTATATGCCACAGCTGTTATAAAAACTGGTACTTGCCCCTTCTCATTTGATAAATTGAATAATTACCTAACTTTTAACAAGTTTTGTTTGTCGTTGAGTCCTACCGGTGCCAACTGTAAGTTTGATGTTGCCGCCCGTACAAGAACCAATGAGCAGGTTGTTAGAAGTTTATATGTAATATATGAAGAAGGAGACAACATAGTGGGTGTACCGTCTGATAATAGTGGTCTTCACGATCTGTCAGTGTTACACTTAGACTCCTGTACAGATTACAATATATATGGTAGAACTGGTGTTGGTATTATTAGACAAACTAACAGCACAATACTTAGTGGCTTATATTACACCTCACTATCAGGTGATTTGTTAGGTTTTAAAAATGTTAGTGATGGTGTTGTCTATTCTGTGACACCATGTGATGTAAGCGCACAAGCGGCTGTTATTGATGGTGCCATAGTTGGAGCTATGACCTCCATTAATAGTGAACTGTTAGGTCTAACACATTGGACAACAACACCTAATTTTTATTACTACTCTATATATAATTACACAAATGAGAGAACTCGTGGCACTGCAATTGACAGTAACGATGTTGATTGTGAACCTATCATAACCTATTCTAACATAGGTGTTTGTAAAAATGGAGCATTGGTCTTTATTAACGTCACACATGCTGATGGAGACGTTCAACCAATTAGCACTGGCAATGTCACGATACCCACAAACTTTACCATATCTGTGCAAGTCGAATACATCCAGGTTTACACCACACCAGTGTCAATAGATTGTTCTAGATACGTTTGTAATGGTAACCCTAGATGTAATAAATTGTTAACACAATATGTCTCTGCATGTCAAACTATTGAGCAAGCGCTTGCAATGAGTGCCAGACTTGAAAACATGGAAGTTGATTCCATGTTGTTTGTTTCAGAAAATGCCCTTAAATTGGCATCTGTTGAGGCGTTCAATAGTACAGAACATTTAGATCCTATTTACAAAGAATGGCCTAACATAGGTGGTTCTTGGCTAGGAGGTCTAAAAGACATACTTCCGTCCCATAATAGCAAACGTAAGTACCGTTCTGCTATAGAAGACTTGCTTTTTGATAAAGTCGTAACTTCTGGTCTAGGTACAGTTGATGAAGATTATAAACGTTGTACAGGTGGTTATGACATAGCTGACTTAGTTTGTGCACAATATTACAATGGCATCATGGTTCTACCTGGTGTGGCTAATGATGACAAGATGACTATGTACACAGCCTCTCTTGCAGGTGGTATAACATTAGGTGCACTAGGTGGTGGCGCCGTGGCTATACCTTTTGCAGTAGCAGTTCAGGCTAGACTTAATTATGTTGCTCTACAAACTGATGTATTGAATAAAAACCAGCAGATCCTGGCTACTGCTTTCAACCAAGCTATTGGTAACATTACACAGGCATTTGGTAAGGTTAATGATGCTATACATCAAACATCACAAGGTCTTGCCACTGTTGCTAAAGCCTTGGCAAAAGTGCAAGATGTTGTTAACACACAAGGGCAAGCTTTAAGCCACCTAACAGTACAACTGCAAAATAACTTTCAAGCCATTAGTAGTTCTATTAGTGACATTTATAATAGGCTTGATGAATTGAGTGCTGATGCACAAGTTGACAGGCTGATTACAGGAAGACTTACAGCACTTAATGCCTTTGTGTCTCAGACTTTAACCAGACAAGCAGAGGTTAGGGCTAGTAGACAACTTGCCAAAGACAAAGTTAATGAATGCGTTAGGTCTCAATCCCAGAGATTTGGATTCTGTGGTAATGGTACACATTTGTTTTCACTTGCAAATGCAGCACCAAATGGCATGATTTTCTTTCACTCAGTACTATTACCAACAGCTTATGAAACTGTGACAGCCTGGTCAGGTATTTGTGCGTCAGATGGCGATCGCACTTTTGGACTTGTAGTTAAGGATGTCCAGCTGACGCTATTCCGCAACCTAGATGACAAGTTTTATTTGACTCCCAGAACTATGTATCAGCCTAGAGCTGCAACTAGTTCTGATTTTGTTCAAATTGAGGGGTGCGATGTGTTGTTTGTCAATGCTACTGTAATTGAATTGCCTAGTATTATACCTGACTACATCGACATTAATCAGACTGTTCAAGACATATTAGAAAACTACAGACCAAACTGGACTGTACCTGAATTGACACTTGACATTTTTAACGCAACCTATTTAAATCTGACTGGTGAAATTGATGACTTAGAATTTAGGTCAGAAAAGCTACATAACACTACTGTAGAACTTGCCATTCTCATTGACAACATTAACAATACATTAGTCAATCTTGAATGGCTCAATAGAATTGAAACTTATGTGAAATGGCCTTGGTATGTGTGGCTACTAATAGGCTTAGTAGTAGTATTTTGCATACCGCTATTGCTATTTTGCTGTTGTAGTACAGGTTGCTGTGGATGCATAGGTTGTTTGGGAAGTTGTTGTCACTCTATTTGTAGTAGAAGACAATTTGAAAATTATGAACCAATTGAAAAAGTGCATGTCCACggagtggctatgcctggcgccgaggatgatgtggtgTAG

1. FCoV strain M91-267 S gene with AcGFP1 tag (S-GFP):GFP tag sequence is indicated by lower case.

ATGATTGTGCTCGTAACTTGCATTTTATTGTTATGCTCATACCACATTGCTTCGAGTACGTCGAATAATGATTGTAGACAAGTTAACGTAACACAATTAGATGGCAATGAAAACCTTATTAAAGACTTTTTGTTTCAAAACTTTAAAGAAGAAGGAAGTGTAGTTGTTGGTGGTTACTACCCGACAGAGGTGTGGTATAACTGTTCTAGGACAGCAACAACTACTGCCTATGAGTATTTCAGTAACATACATGCTTTCTATTTTGATATGGAAGCCATGGAAAATAGTACTGGTAATGCACGGGGTAAACCTTTATTATTCCATGTGCATGGTGAGCCGGTTAGTGTTATCATATATATATCGGCTTATACAAATGATGTGCAACAAAGGCCACTTTTAAAACATGGATTAGTGTGCATAATTAATAGTCGCAATATTGACTATAATAAATTCACCAGTAACCAGTGGGGTTCCATATGTACGGGTAATGACAGAAAAATCCCTTTTTCTGTCATACCCACGGATAATGGAACAAAAATCTACGGGCTTGAGTGGAATGATGAATTTGTTACAGCATACATTAGTGGCCGTTCTTATAATTGGAACATCAATAATAATTGGTTTAACAATGTCACACTTTTTTACTCTCGCTCAAGTACTGCCACATGGCAACACAGTGCTGCATATGTTTACCAAGGTGTTTCTAACTTCACTTATTACAAGTTAAATAACACCAATGGTCTAAAAACCTATGAATTATGTGAAGATTATGAATATTGCACTGGCCACGCCACTAATATCTTTGCCCCAACGGTGGGAGGTTACATACCTGATGGATTTAGTTTTAACAATTGGTTTTTGCTTACAAATAGCTCCACTTTTATTAGTGGCAGATTTGTGACAAATCAGCCATTGTTAGTTAATTGCTTGTGGCCAGTGCCTAGTTTTGGTGTTGCAGCACAAGAATTTTGTTTTGAAGGTGCCCAGTTTAGCCAATGTAATGGTGTGTCTTTAAATAACACAGTGGATGTCATTAGATTCAACCTTAATTTTACTGCAGATGTACAATCTGGCATGGGTGCTACAGTATTTTCACTGAATACAACAGGTGGTGTCATTCTTGAGATTTCTTGTTATAATACAGTGAGCGAGTCAAGTTTTTACAGTTATGGTGAAATTCCATTCGGCGTAACTGATGGACCGCGTTACTGTTATGTACTCCATAATGGCACGGCTCTTAAGTATTTAGGAACATTGCCACCTAGTGTCAAGGAAATTGCTATTAGTAAGTGGGGCCATTTTTATATTAATGGTTACAATTTCTTTAGCACTTTTCCTATTGATTGTATATCTTTCAACTTAACCACTGGTGCTAGTGGAGCTTTCTGGACAATTGCTTACACATCGTACACTGAAGCATTAGTACAAGTTGAAAACACAGCTATTAAGAAGGTGACGTATTGTAACAGTCACATTAATAACATCAAATGTTCTCAACTTACTGCTAATTTGCAAAATGGATTTTATCCTGTTGCCTCAAGTGAAGTAGGTCTCGTTAATAAGAGTGTTGTGTTACTACCTATCTTTCTCACACATACCGCTGTCAATATAACGATTGATCTTGGTATGAAGCGTAGTGGCTACGGTCAACCCATAGCTTCATCATTAAGTAACATCACACTACCGATGCAGGATAATAACACCGATGTGTACTGCATTCGTTCTAACCAATTTTCAGTTTATGTCCACTCCACTTGCAAAAGTTCTTTATGGGACAATATTTTTGATTTAGATTGCACAGATGTTTTATATGCCACAGCTGTTATAAAAACTGGTACTTGCCCCTTCTCATTTGATAAATTGAATAATTACCTAACTTTTAACAAGTTTTGTTTGTCGTTGAGTCCTACCGGTGCCAACTGTAAGTTTGATGTTGCCGCCCGTACAAGAACCAATGAGCAGGTTGTTAGAAGTTTATATGTAATATATGAAGAAGGAGACAACATAGTGGGTGTACCGTCTGATAATAGTGGTCTTCACGATCTGTCAGTGTTACACTTAGACTCCTGTACAGATTACAATATATATGGTAGAACTGGTGTTGGTATTATTAGACAAACTAACAGCACAATACTTAGTGGCTTATATTACACCTCACTATCAGGTGATTTGTTAGGTTTTAAAAATGTTAGTGATGGTGTTGTCTATTCTGTGACACCATGTGATGTAAGCGCACAAGCGGCTGTTATTGATGGTGCCATAGTTGGAGCTATGACCTCCATTAATAGTGAACTGTTAGGTCTAACACATTGGACAACAACACCTAATTTTTATTACTACTCTATATATAATTACACAAATGAGAGAACTCGTGGCACTGCAATTGACAGTAACGATGTTGATTGTGAACCTATCATAACCTATTCTAACATAGGTGTTTGTAAAAATGGAGCATTGGTCTTTATTAACGTCACACATGCTGATGGAGACGTTCAACCAATTAGCACTGGCAATGTCACGATACCCACAAACTTTACCATATCTGTGCAAGTCGAATACATCCAGGTTTACACCACACCAGTGTCAATAGATTGTTCTAGATACGTTTGTAATGGTAACCCTAGATGTAATAAATTGTTAACACAATATGTCTCTGCATGTCAAACTATTGAGCAAGCGCTTGCAATGAGTGCCAGACTTGAAAACATGGAAGTTGATTCCATGTTGTTTGTTTCAGAAAATGCCCTTAAATTGGCATCTGTTGAGGCGTTCAATAGTACAGAACATTTAGATCCTATTTACAAAGAATGGCCTAACATAGGTGGTTCTTGGCTAGGAGGTCTAAAAGACATACTTCCGTCCCATAATAGCAAACGTAAGTACCGTTCTGCTATAGAAGACTTGCTTTTTGATAAAGTCGTAACTTCTGGTCTAGGTACAGTTGATGAAGATTATAAACGTTGTACAGGTGGTTATGACATAGCTGACTTAGTTTGTGCACAATATTACAATGGCATCATGGTTCTACCTGGTGTGGCTAATGATGACAAGATGACTATGTACACAGCCTCTCTTGCAGGTGGTATAACATTAGGTGCACTAGGTGGTGGCGCCGTGGCTATACCTTTTGCAGTAGCAGTTCAGGCTAGACTTAATTATGTTGCTCTACAAACTGATGTATTGAATAAAAACCAGCAGATCCTGGCTACTGCTTTCAACCAAGCTATTGGTAACATTACACAGGCATTTGGTAAGGTTAATGATGCTATACATCAAACATCACAAGGTCTTGCCACTGTTGCTAAAGCCTTGGCAAAAGTGCAAGATGTTGTTAACACACAAGGGCAAGCTTTAAGCCACCTAACAGTACAACTGCAAAATAACTTTCAAGCCATTAGTAGTTCTATTAGTGACATTTATAATAGGCTTGATGAATTGAGTGCTGATGCACAAGTTGACAGGCTGATTACAGGAAGACTTACAGCACTTAATGCCTTTGTGTCTCAGACTTTAACCAGACAAGCAGAGGTTAGGGCTAGTAGACAACTTGCCAAAGACAAAGTTAATGAATGCGTTAGGTCTCAATCCCAGAGATTTGGATTCTGTGGTAATGGTACACATTTGTTTTCACTTGCAAATGCAGCACCAAATGGCATGATTTTCTTTCACTCAGTACTATTACCAACAGCTTATGAAACTGTGACAGCCTGGTCAGGTATTTGTGCGTCAGATGGCGATCGCACTTTTGGACTTGTAGTTAAGGATGTCCAGCTGACGCTATTCCGCAACCTAGATGACAAGTTTTATTTGACTCCCAGAACTATGTATCAGCCTAGAGCTGCAACTAGTTCTGATTTTGTTCAAATTGAGGGGTGCGATGTGTTGTTTGTCAATGCTACTGTAATTGAATTGCCTAGTATTATACCTGACTACATCGACATTAATCAGACTGTTCAAGACATATTAGAAAACTACAGACCAAACTGGACTGTACCTGAATTGACACTTGACATTTTTAACGCAACCTATTTAAATCTGACTGGTGAAATTGATGACTTAGAATTTAGGTCAGAAAAGCTACATAACACTACTGTAGAACTTGCCATTCTCATTGACAACATTAACAATACATTAGTCAATCTTGAATGGCTCAATAGAATTGAAACTTATGTGAAATGGCCTTGGTATGTGTGGCTACTAATAGGCTTAGTAGTAGTATTTTGCATACCGCTATTGCTATTTTGCTGTTGTAGTACAGGTTGCTGTGGATGCATAGGTTGTTTGGGAAGTTGTTGTCACTCTATTTGTAGTAGAAGACAATTTGAAAATTATGAACCAATTGAAAAAGTGCATGTCCACgtgagcaagggcgccgagctgttcaccggcatcgtgcccatcctgatcgagctgaatggcgatgtgaatggccacaagttcagcgtgagcggcgagggcgagggcgatgccacctacggcaagctgaccctgaagttcatctgcaccaccggcaagctgcctgtgccctggcccaccctggtgaccaccctgagctacggcgtgcagtgcttctcacgctaccccgatcacatgaagcagcacgacttcttcaagagcgccatgcctgagggctacatccaggagcgcaccatcttcttcgaggatgacggcaactacaagtcgcgcgccgaggtgaagttcgagggcgataccctggtgaatcgcatcgagctgaccggcaccgatttcaaggaggatggcaacatcctgggcaataagatggagtacaactacaacgcccacaatgtgtacatcatgaccgacaaggccaagaatggcatcaaggtgaacttcaagatccgccacaacatcgaggatggcagcgtgcagctggccgaccactaccagcagaatacccccatcggcgatggccctgtgctgctgcccgataaccactacctgtccacccagagcgccctgtccaaggaccccaacgagaagcgcgatcacatgatctacttcggcttcgtgaccgccgccgccatcacccacggcatggatgagctgtacaagTGA

1. SARS-CoV-2 S

ATGTTTGTCTTCCTGGTGCTTTTGCCCCTCGTTAGTTCACAGTGTGTGAACCTTACTACTAGAACCCAACTGCCTCCCGCTTATACTAATAGTTTCACCCGGGGCGTGTACTATCCAGACAAGGTCTTTAGGTCCAGTGTGCTGCATTCCACACAGGACCTGTTTCTTCCGTTCTTTTCCAACGTGACATGGTTTCACGCAATTCACGTGTCTGGGACCAACGGCACCAAAAGATTCGATAATCCAGTCCTCCCCTTCAATGACGGGGTGTATTTTGCCAGCACAGAGAAGTCAAATATCATCCGAGGCTGGATTTTCGGCACAACTCTCGATTCCAAAACTCAATCTCTTCTCATTGTCAATAACGCCACCAATGTTGTGATCAAAGTGTGCGAATTCCAGTTTTGTAATGACCCCTTTCTGGGTGTCTATTACCATAAGAACAATAAAAGCTGGATGGAATCTGAATTCCGAGTTTACTCATCTGCAAACAATTGCACATTTGAGTATGTCAGTCAACCGTTCTTGATGGATCTGGAGGGCAAACAAGGAAATTTCAAGAATCTGCGGGAGTTTGTGTTCAAAAACATTGACGGCTATTTTAAGATCTATTCTAAACACACACCTATCAACCTTGTCAGGGACCTGCCCCAGGGGTTTTCAGCTCTGGAGCCTCTGGTGGATCTGCCAATCGGCATCAACATCACACGGTTTCAGACCCTTTTGGCGCTGCATAGGAGTTACCTCACCCCAGGAGATTCAAGCAGTGGCTGGACAGCTGGTGCAGCAGCTTATTACGTGGGATACCTGCAACCACGGACTTTTCTGCTCAAATACAATGAGAACGGTACTATAACCGATGCTGTGGACTGTGCTCTTGATCCACTGAGCGAGACCAAGTGCACTCTTAAAAGCTTCACCGTGGAGAAGGGCATTTATCAGACCTCCAACTTCAGGGTCCAGCCCACTGAATCCATCGTCAGATTCCCAAATATTACCAACCTGTGTCCATTCGGAGAAGTGTTCAACGCCACTCGGTTTGCTAGCGTATATGCTTGGAATCGCAAGAGGATTTCAAATTGTGTGGCTGACTATTCTGTGCTGTACAATTCCGCGTCATTTAGCACATTCAAGTGTTACGGTGTTAGCCCCACAAAACTCAACGATCTGTGTTTCACCAATGTGTATGCCGACAGTTTCGTTATAAGGGGCGATGAAGTGAGACAAATTGCCCCTGGACAGACTGGAAAGATTGCGGATTACAATTACAAACTCCCAGACGATTTCACAGGGTGTGTTATTGCATGGAATAGCAATAATCTTGATAGTAAGGTCGGGGGGAATTATAATTATCTGTATCGGCTGTTCAGAAAGTCTAACCTGAAACCCTTTGAGAGGGATATCAGTACTGAAATTTACCAGGCGGGTTCTACTCCATGCAATGGGGTGGAGGGGTTTAACTGCTATTTTCCACTGCAGTCCTACGGCTTCCAGCCAACAAATGGCGTGGGCTACCAACCCTACAGGGTGGTTGTGCTCAGCTTTGAGTTGCTGCATGCCCCTGCCACTGTGTGTGGGCCAAAAAAGTCCACTAACCTGGTGAAAAACAAATGTGTCAATTTTAATTTCAACGGCCTGACTGGCACCGGAGTGTTGACTGAATCCAATAAAAAATTCCTCCCATTTCAGCAATTCGGAAGAGATATAGCGGATACTACCGACGCCGTTAGGGATCCACAGACCTTGGAGATCCTCGATATTACCCCATGCTCCTTTGGAGGGGTGTCCGTGATTACCCCTGGCACTAATACCAGTAATCAGGTGGCTGTGCTGTACCAGGACGTAAACTGCACGGAGGTCCCTGTAGCCATTCACGCAGACCAACTGACGCCTACCTGGAGGGTGTACTCAACGGGTTCTAATGTCTTCCAGACTAGAGCGGGCTGTCTGATCGGTGCGGAGCATGTTAATAACTCCTATGAGTGCGACATTCCTATCGGGGCTGGAATCTGTGCCAGCTATCAGACACAGACTAACAGTCCGAGAAGGGCCAGGTCCGTGGCCTCTCAATCCATCATCGCCTACACAATGTCTCTCGGCGCCGAAAATAGCGTGGCTTATTCAAACAACTCAATCGCCATCCCAACAAATTTCACCATCTCCGTGACTACCGAGATCCTTCCTGTATCCATGACCAAGACGAGTGTGGATTGCACAATGTATATTTGCGGCGACTCAACGGAGTGCTCCAACCTGCTGCTGCAGTACGGGTCCTTCTGCACTCAGCTTAATCGCGCTCTGACAGGGATCGCCGTCGAACAGGACAAAAACACCCAAGAGGTGTTTGCCCAGGTTAAGCAGATCTATAAGACCCCCCCCATTAAAGACTTTGGAGGGTTCAACTTTTCCCAGATCCTTCCTGATCCTTCTAAGCCCAGCAAAAGGTCCTTCATCGAGGACCTGCTGTTTAACAAAGTTACCCTCGCCGATGCCGGCTTCATTAAGCAGTATGGCGATTGCCTGGGCGACATTGCTGCTCGCGACCTTATCTGCGCACAAAAATTTAACGGGCTGACTGTGCTGCCGCCTTTGCTGACCGACGAGATGATCGCCCAATACACTTCCGCCCTGCTCGCTGGGACCATTACCTCTGGCTGGACTTTTGGAGCGGGCGCTGCTCTTCAGATTCCCTTTGCCATGCAGATGGCCTACAGATTCAATGGCATAGGCGTGACGCAGAACGTACTGTACGAAAACCAGAAACTCATAGCTAATCAGTTCAATAGCGCGATAGGAAAAATCCAAGATTCTCTGAGCTCTACCGCCTCAGCTCTGGGCAAACTTCAGGATGTTGTTAATCAGAATGCTCAGGCCCTGAACACTCTGGTGAAGCAGCTCTCCAGTAATTTCGGCGCAATCTCCAGCGTTCTGAACGACATATTGAGCCGCCTTGATAAGGTCGAGGCAGAAGTCCAGATTGACCGGCTGATTACTGGACGGCTGCAGTCCCTGCAGACATACGTGACCCAGCAACTCATTCGGGCCGCCGAAATTCGCGCGAGCGCAAATCTTGCTGCCACCAAAATGAGCGAGTGCGTTTTGGGACAGAGTAAGCGCGTGGACTTCTGCGGCAAGGGTTACCATCTGATGTCTTTCCCCCAGTCTGCACCTCACGGAGTGGTGTTCCTGCACGTGACTTACGTGCCTGCCCAGGAGAAGAACTTCACAACCGCACCAGCCATCTGTCACGACGGTAAAGCCCATTTTCCCCGCGAAGGGGTGTTTGTTAGTAATGGGACCCATTGGTTTGTGACCCAGCGGAATTTCTATGAACCACAGATCATTACCACAGATAACACGTTCGTGAGCGGGAACTGCGACGTAGTGATCGGAATCGTGAACAATACAGTTTACGATCCCCTGCAGCCGGAGCTGGACTCCTTTAAGGAAGAGCTTGACAAATACTTCAAAAACCACACCAGCCCTGACGTGGATTTGGGGGATATTTCAGGAATCAATGCCTCCGTGGTTAACATTCAGAAAGAAATAGATAGACTGAACGAAGTCGCTAAGAATCTGAACGAGTCCCTGATCGACCTGCAGGAACTTGGGAAGTATGAGCAATATATCAAATGGCCTTGGTACATTTGGCTCGGCTTCATCGCAGGCCTGATCGCAATTGTCATGGTTACGATAATGTTGTGTTGCATGACGTCCTGTTGCTCATGTTTGAAAGGTTGCTGCAGCTGTGGGTCCTGTTGCAAGTTTGATGAGGACGACAGTGAGCCAGTGCTTAAAGGAGTTAAACTCCACTACACGTGA

1. Human ACE2

ATGAGCTCTTCATCCTGGCTGCTCTTGAGCTTGGTGGCTGTAACGGCTGCGCAGTCCACAATTGAAGAACAAGCCAAGACATTCTTGGACAAATTCAACCATGAAGCCGAAGACTTGTTCTATCAGAGCTCACTTGCATCATGGAACTACAATACCAATATTACCGAGGAGAATGTACAGAATATGAATAATGCAGGAGACAAGTGGAGCGCCTTTCTCAAGGAACAGTCTACTCTGGCTCAGATGTACCCCCTCCAGGAAATCCAGAACCTCACAGTGAAGTTGCAGCTGCAGGCCCTCCAACAAAATGGGTCCTCCGTTCTGTCAGAAGATAAGAGTAAGAGACTGAACACCATTCTGAATACCATGTCAACAATCTACAGCACCGGAAAGGTCTGCAATCCAGATAACCCACAGGAATGTTTGCTTCTTGAGCCGGGACTCAACGAAATCATGGCAAATTCTCTTGATTACAATGAGAGGCTGTGGGCATGGGAATCCTGGAGGTCAGAGGTGGGTAAGCAGCTGCGGCCTCTGTACGAGGAATACGTGGTACTGAAGAACGAGATGGCAAGGGCCAACCATTACGAGGACTATGGCGACTACTGGCGCGGGGACTACGAAGTGAACGGCGTTGATGGGTACGACTACAGCAGAGGACAATTGATCGAAGACGTAGAACATACGTTTGAGGAGATCAAGCCATTGTACGAACACCTGCATGCTTACGTGCGCGCAAAGCTGATGAACGCTTATCCAAGCTACATCAGCCCGATTGGATGCCTGCCAGCTCACTTGCTCGGAGATATGTGGGGCCGCTTCTGGACGAACCTGTACAGCCTTACAGTCCCATTTGGGCAAAAGCCTAATATTGACGTCACCGATGCAATGGTTGATCAGGCCTGGGATGCCCAGCGAATTTTCAAAGAAGCTGAAAAGTTCTTCGTGTCTGTGGGGTTGCCTAACATGACCCAGGGTTTCTGGGAGAATTCTATGCTGACTGACCCTGGAAATGTACAGAAGGCAGTTTGCCATCCCACAGCCTGGGACCTCGGGAAAGGGGACTTCCGCATTCTTATGTGTACAAAGGTGACCATGGATGACTTCTTGACTGCACACCACGAGATGGGCCACATTCAGTATGACATGGCTTACGCTGCGCAGCCATTCCTCCTTAGAAATGGAGCCAATGAAGGGTTCCACGAGGCCGTGGGCGAGATTATGTCCCTGAGCGCCGCTACCCCAAAACACCTGAAAAGCATAGGTTTGTTGAGCCCCGATTTTCAAGAAGACAACGAAACAGAAATCAACTTCCTGCTTAAACAGGCCCTTACCATTGTTGGGACTCTGCCATTTACATACATGCTGGAGAAATGGAGATGGATGGTATTCAAGGGGGAGATCCCCAAGGATCAGTGGATGAAAAAGTGGTGGGAGATGAAGCGGGAAATTGTCGGGGTTGTAGAACCAGTCCCACATGATGAGACATACTGCGACCCCGCCTCTCTGTTCCACGTGTCCAACGATTACTCATTCATTCGATATTACACCAGGACCCTGTATCAGTTCCAGTTTCAGGAGGCCCTCTGTCAGGCCGCAAAGCACGAGGGCCCCCTGCACAAGTGCGATATTAGTAATTCCACTGAGGCTGGACAGAAGCTGTTCAATATGCTTCGCCTCGGCAAGAGTGAACCCTGGACCCTGGCCCTGGAAAACGTAGTAGGGGCAAAGAACATGAACGTTCGGCCTCTGCTGAACTACTTCGAACCCCTTTTCACTTGGCTCAAGGATCAGAACAAGAACTCATTCGTCGGATGGTCTACTGACTGGTCCCCATATGCCGACCAGAGCATTAAAGTGAGAATTAGCCTCAAGAGTGCTCTTGGCGATAAAGCTTATGAGTGGAATGATAATGAGATGTACCTGTTTCGCTCCAGCGTGGCCTATGCAATGCGACAGTACTTCCTCAAGGTGAAGAACCAGATGATTCTGTTCGGCGAGGAAGACGTGCGAGTGGCGAACTTGAAGCCTAGAATCAGCTTTAATTTCTTCGTGACCGCACCTAAGAATGTGTCCGATATCATCCCACGAACTGAGGTTGAAAAGGCCATACGGATGAGTCGGTCCAGAATTAATGATGCCTTCCGCCTTAACGACAATAGCCTGGAGTTCTTGGGTATTCAACCTACGCTGGGCCCACCCAATCAGCCACCTGTGTCTATTTGGCTGATTGTGTTTGGGGTGGTGATGGGGGTGATTGTGGTCGGCATCGTGATCCTGATTTTTACTGGCATTAGAGATCGAAAAAAAAAGAATAAAGCACGCTCCGGTGAGAATCCTTATGCCTCAATAGACATCTCCAAGGGGGAAAATAACCCTGGTTTCCAAAATACAGACGATGTGCAAACCTCTTGA

1. Feline ACE2

ATGTCAGGCTCTTTCTGGCTCCTTCTCAGCTTTGCTGCTTTAACTGCTGCTCAATCCACCACTGAAGAACTGGCCAAGACATTTTTGGAGAAGTTTAACCATGAAGCCGAAGAGCTGTCTTATCAAAGTTCACTTGCTTCCTGGAATTATAACACCAACATCACAGACGAGAATGTCCAAAAAATGAATGAGGCCGGGGCCAAATGGTCTGCCTTTTATGAAGAACAGTCCAAGCTTGCCAAAACTTACCCGCTAGCAGAAATTCACAATACCACCGTCAAACGTCAATTGCAGGCCCTTCAGCAGAGTGGGTCATCAGTGCTCTCAGCAGACAAGAGCCAACGATTGAACACAATCTTAAATGCAATGAGCACGATCTACAGTACTGGAAAAGCTTGTAACCCAAACAATCCACAGGAGTGCTTATTACTTGAACCAGGCTTGGATGACATCATGGAAAACAGCAAAGACTACAACGAGAGGCTCTGGGCTTGGGAAGGCTGGAGGGCTGAGGTCGGCAAGCAGCTGAGGCCATTATATGAAGAGTACGTGGCCCTGAAAAATGAGATGGCAAGAGCAAACAATTATGAGGACTATGGAGATTATTGGAGAGGAGATTATGAAGAGGAGTGGACAGATGGCTATAACTATAGCCGCAGCCAGTTGATTAAAGACGTGGAACATACCTTCACACAGATTAAGCCACTGTACCAACATCTTCATGCTTACGTGAGGGCAAAGTTGATGGATACCTACCCTTCCCGTATCAGCCCAACTGGATGCCTCCCTGCCCATTTGCTTGGCGATATGTGGGGTCGATTTTGGACAAATCTGTACCCTTTGACAGTCCCCTTTGGACAGAAACCAAACATAGATGTTACTGATGCAATGGTGAACCAGAGCTGGGATGCAAGGAGGATATTCAAGGAAGCTGAGAAATTCTTTGTGTCTGTTGGCCTTCCCAACATGACTCAAGGATTCTGGGAAAACTCCATGCTAACCGAGCCAGGAGACAGCCGGAAAGTGGTCTGCCACCCCACAGCTTGGGACCTAGGGAAGGGTGACTTCAGGATCAAGATGTGCACAAAGGTGACGATGGATGACTTCCTGACGGCCCATCACGAGATGGGACACATCCAGTATGACATGGCATATGCCGTGCAACCCTTCCTGCTAAGAAATGGAGCTAATGAGGGGTTCCATGAAGCTGTCGGGGAGATCATGTCACTTTCTGCGGCTACACCCAACCATCTGAAAACCATTGGTCTTCTGTCACCTGGTTTTTCTGAAGACAGTGAAACAGAAATAAACTTCCTACTCAAACAAGCACTTACAATTGTTGGAACGCTACCGTTTACTTATATGTTAGAAAAGTGGAGGTGGATGGTCTTTAAGGGTGAAATTCCCAAGGAGCAGTGGATGCAAAAGTGGTGGGAGATGAAGCGAGAGATAGTCGGGGTGGTGGAGCCTGTGCCCCATGATGAAACATACTGTGACCCTGCATCTCTGTTCCATGTTGCTAATGATTACTCATTCATCAGATACTACACAAGGACCATTTATCAATTCCAGTTTCAAGAAGCCCTTTGTCGAATAGCTAAACATGAAGGTCCCCTGCACAAATGTGATATCTCAAATTCCAGTGAAGCCGGGAAGAAGCTGCTCCAAATGCTGACCCTTGGAAAATCAAAGCCCTGGACCTTAGCATTGGAACATGTTGTAGGAGAAAAGAAAATGAATGTAACACCACTGCTCAAGTACTTTGAGCCCTTGTTTACCTGGCTGAAAGAGCAGAACAGGAATTCTTTTGTGGGATGGAACACTGACTGGCGTCCATATGCTGACCAAAGCATTAAAGTGAGGATAAGCCTAAAATCAGCTCTTGGAGACGAAGCATATGAATGGAATGACAATGAAATGTACTTGTTCCGGTCATCTGTTGCATACGCCATGAGAGAATATTTTTCCAAAGTCAAAAACCAGACGATTCCTTTTGTGGAGGATAACGTGTGGGTGAGCAATTTGAAACCAAGGATCTCCTTCAACTTCTTTGTCACTGCATCGAAAAATGTGTCTGACGTCATTCCTAGAAGTGAAGTCGAAGAGGCCATCAGGATGTCCCGGAGCCGTATCAATGATGCTTTCCGCCTGGATGACAACAGCCTGGAGTTTCTGGGTATTCAGCCAACTCTGTCACCCCCTTACCAGCCACCTGTCACCATATGGCTGATTGTTTTTGGGGTCGTGATGGGTGTGGTAGTGGTTGGTATTGTCCTGCTCATCGTCTCCGGGATCAGAAATCGAAGGAAGAACAATCAAGCAAGAAGTGAAGAAAATCCTTACGCCTCCGTGGACTTGAGTAAAGGAGAAAATAATCCAGGATTCCAACATGCTGATGATGTTCAGACTTCATTTTAG
